# The Dynamic Genome and Transcriptome of the Human Fungal Pathogen *Blastomyces* and Close Relative *Emmonsia*

**DOI:** 10.1371/journal.pgen.1005493

**Published:** 2015-10-06

**Authors:** José F. Muñoz, Gregory M. Gauthier, Christopher A. Desjardins, Juan E. Gallo, Jason Holder, Thomas D. Sullivan, Amber J. Marty, John C. Carmen, Zehua Chen, Li Ding, Sharvari Gujja, Vincent Magrini, Elizabeth Misas, Makedonka Mitreva, Margaret Priest, Sakina Saif, Emily A. Whiston, Sarah Young, Qiandong Zeng, William E. Goldman, Elaine R. Mardis, John W. Taylor, Juan G. McEwen, Oliver K. Clay, Bruce S. Klein, Christina A. Cuomo

**Affiliations:** 1 Cellular and Molecular Biology Unit, Corporación para Investigaciones Biológicas, Medellín, Colombia; 2 Institute of Biology, Universidad de Antioquia, Medellín, Colombia; 3 Department of Medicine, University of Wisconsin, Madison, Madison, Wisconsin, United States of America; 4 Broad Institute of MIT and Harvard, Cambridge, Massachusetts, United States of America; 5 Doctoral Program in Biomedical Sciences, Universidad del Rosario, Bogotá, Colombia; 6 Department of Pediatrics, University of Wisconsin, Madison, Madison, Wisconsin, United States of America; 7 The Genome Institute, Washington University School of Medicine, St. Louis, Missouri, United States of America; 8 Department of Plant and Microbial Biology, University of California, Berkeley, Berkeley, California, United States of America; 9 Department of Microbiology and Immunology, School of Medicine, University of North Carolina, Chapel Hill, Chapel Hill, North Carolina, United States of America; 10 School of Medicine, Universidad de Antioquia, Medellín, Colombia; 11 School of Medicine and Health Sciences, Universidad del Rosario, Bogotá, Colombia; 12 Department of Medical Microbiology & Immunology, University of Wisconsin, Madison, Madison, Wisconsin, United States of America; DOE Joint Genome Institute, UNITED STATES

## Abstract

Three closely related thermally dimorphic pathogens are causal agents of major fungal diseases affecting humans in the Americas: blastomycosis, histoplasmosis and paracoccidioidomycosis. Here we report the genome sequence and analysis of four strains of the etiological agent of blastomycosis, *Blastomyces*, and two species of the related genus *Emmonsia*, typically pathogens of small mammals. Compared to related species, *Blastomyces* genomes are highly expanded, with long, often sharply demarcated tracts of low GC-content sequence. These GC-poor isochore-like regions are enriched for *gypsy* elements, are variable in total size between isolates, and are least expanded in the avirulent *B*. *dermatitidis* strain ER-3 as compared with the virulent *B*. *gilchristii* strain SLH14081. The lack of similar regions in related species suggests these isochore-like regions originated recently in the ancestor of the *Blastomyces* lineage. While gene content is highly conserved between *Blastomyces* and related fungi, we identified changes in copy number of genes potentially involved in host interaction, including proteases and characterized antigens. In addition, we studied gene expression changes of *B*. *dermatitidis* during the interaction of the infectious yeast form with macrophages and in a mouse model. Both experiments highlight a strong antioxidant defense response in *Blastomyces*, and upregulation of dioxygenases *in vivo* suggests that dioxide produced by antioxidants may be further utilized for amino acid metabolism. We identify a number of functional categories upregulated exclusively *in vivo*, such as secreted proteins, zinc acquisition proteins, and cysteine and tryptophan metabolism, which may include critical virulence factors missed before in *in vitro* studies. Across the dimorphic fungi, loss of certain zinc acquisition genes and differences in amino acid metabolism suggest unique adaptations of *Blastomyces* to its host environment. These results reveal the dynamics of genome evolution and of factors contributing to virulence in *Blastomyces*.

## Author Summary

Dimorphic fungal pathogens including *Blastomyces* are the cause of major fungal diseases in North and South America. The genus *Emmonsia* includes species infecting small mammals as well as a newly emerging pathogenic species recently reported in HIV-positive patients in South Africa. Here, we synthesize both genome sequencing of four isolates of *Blastomyces* and two species of *Emmonsia* as well as deep sequencing of *Blastomyces* RNA to draw major new insights into the evolution of this group and the pathogen response to infection. We investigate the trajectory of genome evolution of this group, characterizing the phylogenetic relationships of these species, a remarkable genome expansion that formed large isochore-like regions of low GC content in *Blastomyces*, and variation of gene content, related to host interaction, among the dimorphic fungal pathogens. Using RNA-Seq, we profile the response of *Blastomyces* to macrophage and mouse pulmonary infection, identifying key pathways and novel virulence factors. The identification of key fungal genes involved in adaptation to the host suggests targets for further study and therapeutic intervention in *Blastomyces* and related dimorphic fungal pathogens.

## Introduction

*Blastomyces* is a genus of a thermally dimorphic fungal pathogen, which is the etiological agent of blastomycosis, a lung infection that can become a systemic mycosis. In North America, *Blastomyces* is endemic in the Ohio and Mississippi river valleys, the Great Lakes region, and the St. Lawrence River [1]. Within *Blastomyces*, two lineages of *B*. *dermatitidis* have been recognized [2], with recent work providing evidence that one lineage is a distinct species, *B*. *gilchristii* [3]. Both species can infect humans, and vary in morphology, virulence and immune responses by the host. The primary mode of infection is inhalation of conidia and the subsequent conversion of these conidia into parasitic yeast [4,5]. Clinical manifestations range from asymptomatic infection to symptomatic disease and include pneumonia, acute respiratory distress syndrome, and a rapidly progressive dissemination involving multiple organ systems that is often fatal [5,6]. Diagnosis is often complicated by the similarity of symptoms to those of viral or bacterial respiratory infection and by the aforementioned variety of manifestations [7].

As a thermally dimorphic fungus, *Blastomyces* has the remarkable ability to switch between two different morphologies in response to external stimuli, predominantly temperature [5]. At 22–25°C, *Blastomyces* grows as septate hyphae that produce infectious conidia and at 37°C it grows as a budding yeast [8]. *Blastomyces* is part of a larger group of dimorphic fungal pathogens, including *Histoplasma*, *Paracoccidioides*, and *Coccidioides*, all belonging to the order Onygenales. The dimorphic fungi collectively are the most common cause of invasive fungal disease worldwide and account for several million infections each year [8]. Unlike opportunistic fungi, such as *Candida albicans*, *Cryptococcus neoformans*, or *Aspergillus fumigatus*, the dimorphic fungi can infect immunocompetent and immunocompromised hosts [6,9–11].

Previous work has shown that in *Blastomyces*, the temperature-dependent switch from hyphae to yeast along with upregulation of yeast-phase specific genes is critical for virulence [12–14]. The dimorphism-regulating kinase-1 (*DRK1*) promotes the temperature-dependent conversion from mold to yeast, and its deletion renders *Blastomyces* avirulent during experimental murine pulmonary infection [12]. The upregulation of yeast-phase specific genes, such as the *Blastomyces* yeast-phase specific gene 1 (*BYS1*) [15] and the *Blastomyces* adhesion-1 gene (*BAD1*) [13,14], is also important for the adaptive response of the yeast cells in the host environment. *BAD1* is considered an essential virulence factor in *Blastomyces*, since it binds yeast cells to host tissue and impairs host immune defenses by inhibiting the production of tumor necrosis factor-α and blocking CD4^+^ T lymphocyte activation [13].

Within the Onygenales, *Blastomyces*, *Histoplasma* and *Paracoccidioides* belong to the family Ajellomycetaceae. Also within Ajellomycetaceae is the genus *Emmonsia*, which includes *E*. *crescens* and *E*. *parva*, the etiological agents of adiaspiromycosis, a pulmonary disease of small mammals and occasionally of humans [16]. Recently, a cluster of systemic infections of HIV-positive patients in South Africa were shown to be caused by *Emmonsia* isolates [17]. While *E*. *crescens* and *E*. *parva* also undergo a dimorphic shift at high temperature, they transform into large, thick-walled adiaspores rather than yeast cells [18] (S1 Table). Two phylogenetic studies using 18S ribosomal DNA sequences found that *E*. *parva* was the sister species to *Blastomyces* [19,20]. The positioning of *E*. *crescens* was less clear; in one analysis it was a sister group to *Paracoccidioides* [19] while in the other analysis it was grouped with *Blastomyces* and *E*. *parva* [20]. In neither phylogeny was the alternative positioning of *E*. *crescens* strongly supported.

To further investigate the genomic basis of differences observed among the Ajellomycetaceae in terms of pathogenicity, morphology, and the infection process, we sequenced six genomes of *Blastomyces* and *Emmonsia*, as well as sequencing the *B*. *dermatitidis* transcriptome during macrophage co-cultivation and *in vivo* pulmonary infection. The newly sequenced genomes included three representative strains of *B*. *dermatitidis* (ER-3, ATCC18188, and ATCC26199), and one strain of each of *B*. *gilchristii* (SLH14081), *E*. *parva* (UAMH139), and *E*. *crescens* (UAMH3008). *Blastomyces dermatitidis* ER-3 was isolated from a woodpile located in a highly endemic region of Wisconsin and is hypovirulent in mice [21,22]. The ATCC18188 strain is the only current example of the 'a' mating type (*MAT1-1* locus) available for *B*. *dermatitidis* [23]. ATCC26199 is a clinical isolate from South Carolina that is commonly used for *in vitro* and *in vivo* laboratory assays [14]. *Blastomyces gilchristii* SLH14081 is a human clinical isolate that is highly virulent in a murine model of blastomycosis [22,24]. Both *Emmonsia* strains were isolated from small mammals, *E*. *parva* from a weasel in Ravelli County, Montana, and *E*. *crescens* from lungs of a rodent (*Arvicola terrestris*) in Norway.

Utilizing this genomic data, we find that the *Blastomyces* genomes are much larger than those of their close relatives, and are characterized by large, isochore-like GC-poor regions overrun by repetitive elements. Our whole-genome analyses provide further evidence for the phylogenetic relationships between *Blastomyces* and *Emmonsia* and other Onygenales. Finally, we identify novel sets of candidate virulence factors through comparison of the *Blastomyces* transcription during *in vivo* pulmonary infection to growth in co-culture with macrophages or in different media or temperature. This combination of genomic and transcriptomic analysis provides a foundation and new candidate genes to further characterize the underlying molecular differences that determine the infectious potency of *Blastomyces* strains and give rise to the clinical profiles attributable to blastomycosis.

## Results

### Expanded genomes of *Blastomyces* species

**Fig 1:**
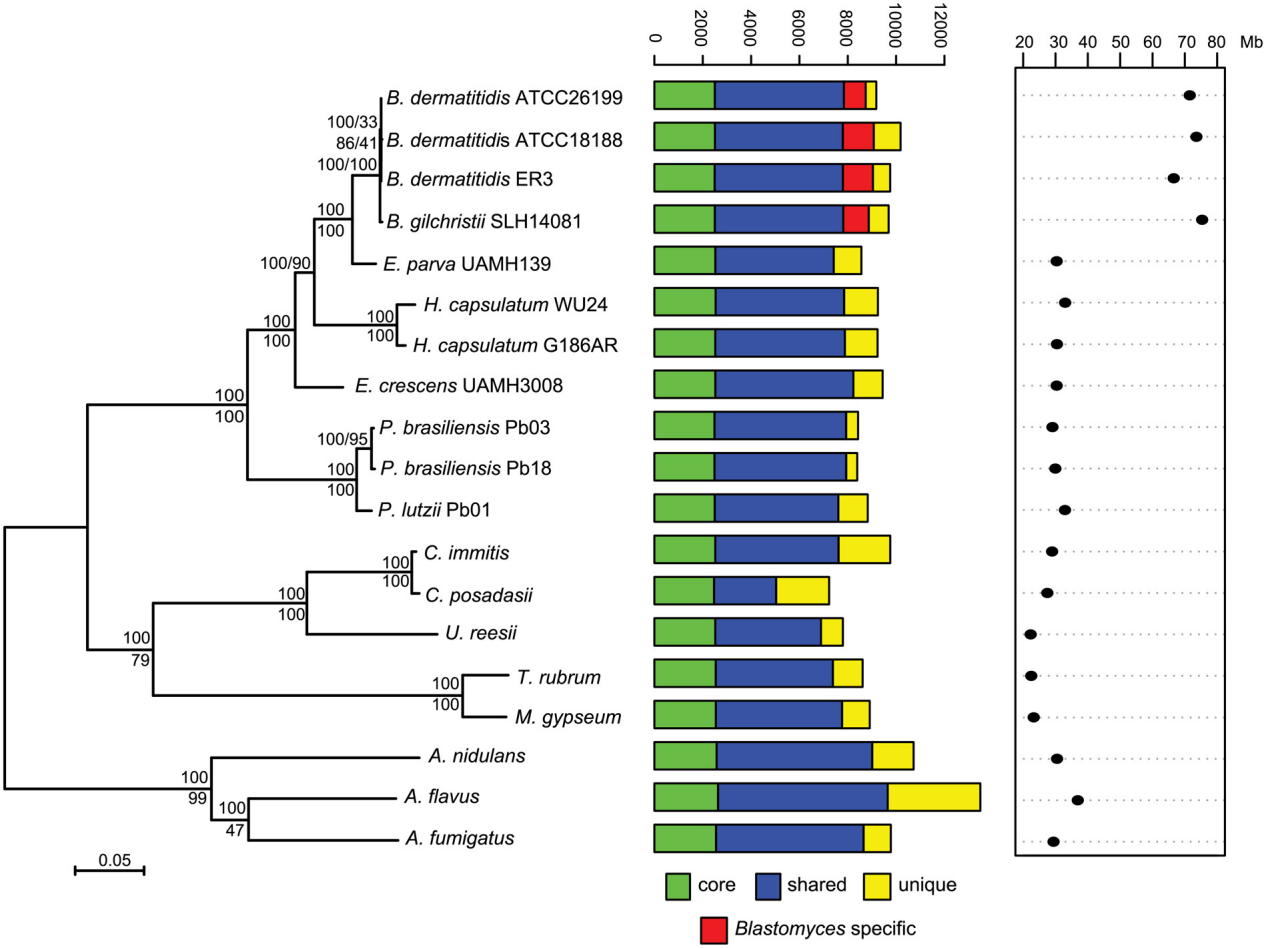
Phylogeny and gene conservation of *Blastomyces* and *Emmonsia* spp. Maximum likelihood tree inferred from concatenated protein alignments of 2,062 core genes based on 1,000 replicates; all bootstrap values (top value for each node) were 100% except for one node within *B*. *dermatitidis*, which was 88%. Branch order was also well supported by the consensus of individual gene trees (GSF, lower value for each node). A bar plot of orthology classes is shown to the right, where core genes found in all genomes are shown in green, shared genes present in more than one but not all genomes in blue, genes specific to *Blastomyces* genomes in red, and genes that were unique to only one of the 19 genomes in yellow. Finally, genome size is plotted for each genome along the x-axis, which ranges from 20 to 80 Mb.

We sequenced and assembled the genomes of three *Blastomyces dermatitidis* strains and one *B*. *gilchristii* strain, and representatives of two *Emmonsia* species. The *Blastomyces* strains were sequenced using either Sanger technology or a hybrid of Sanger and 454 technologies. The *Emmonsia* strains were sequenced using Illumina technology, and *de novo* assemblies were generated for each strain (Methods). Comparison of the genomes of four *Blastomyces* strains, SLH14081, ER-3, ATCC18188 and ATCC26199, revealed they were over twice the size of all other Onygenales. The *Blastomyces* assemblies range in size from 66.6 Mb for *B*. *dermatitidis* strain ER-3 to 75.4 Mb *B*. *gilchristii* strain SLH14081 (Table 1). These assemblies were over twice as large as those of other dimorphic pathogens in the order Onygenales including the *Emmonsia* species (30.4 Mb), although the use of only short reads from a single library for the two *Emmonsia* may under-represent repetitive sequence (Fig 1). The assemblies of two *Blastomyces* strains, SLH14081 and ER-3, were sequenced to a higher depth than the other two strains, and as a result contain nearly all of the assembled sequence in a relatively small number of scaffolds, 100 and 25 scaffolds respectively. As an independent assessment of genome size and structure, we generated an optical map of the SLH14081 strain (S1 Fig). Consistent with our assembly of this strain, the map had an estimated size of 79.6 Mb, arranged in eighteen linkage groups. In addition, a total of 65.9 Mb of the 75.4 Mb of the SLH14081 assembly was anchored to the optical map (S2 Table).

**Table 1:** Assembly and annotation statistics for *Blastomyces* and *Emmonsia* genomes. *Bd*: *B*. *dermatitidis*, *Bg*: *B*. *gilchristii*, *Ep*: *E*. *parva*, *Ec*: *E*. *crescens*.

	Total assembly length	Scaffolds	Scaffold N50	GC-content (%)	Genes	Coding (%)	Intergenic length	Repeat (%)
*Bg* SLH14081	75.35 Mb	100	2.44 Mb	35.8	9,692	16.9	7.2 kb	63.0
*Bd* ER-3	66.57 Mb	25	5.55 Mb	37.1	9,755	19.2	6.0 kb	60.0
*Bd* ATCC18188	73.58 Mb	4,159	0.40 Mb	36.7	10,187	17.4	4.2 kb	56.6
*Bd* ATCC26199	71.52 Mb	3,282	0.29 Mb	36.6	9,180	17.5	4.5 kb	58.5
*Ep* UAMH139	30.35 Mb	2,682	31.17 kb	44.7	8,563	35.6	1.4 kb	9.9
*Ec* UAMH3008	30.36 Mb	1,150	95.15 kb	45.4	9,444	41.8	1.4 kb	5.4

The total number of predicted genes in *Blastomyces*, *Emmonsia*, and other related fungi was similar despite the large difference in genome size. In *Blastomyces*, the number of predicted genes varied between 9,180 in ATCC26199 to 10,187 in ATCC18188; for *E*. *parva* and *E*. *crescens* the counts were similar, 8,563 and 9,444, respectively (Table 1), as were those of other sequenced Onygenales (Fig 1). High representation of core eukaryotic genes in each genome provides evidence that their gene sets are nearly complete; *E*. *parva* includes 88% of core eukaryotic genes, while the *E*. *crescens* and *Blastomyces* gene sets include 96–98% (S2 Fig).

### Phylogenetic position of *Blastomyces*, *Emmonsia parva* and *E*. *crescens*

To compare gene content and conservation, we identified orthologous gene clusters in the six genomes sequenced here, 10 additional Onygenales genomes, including three other pathogenic species (*Histoplasma*, *Paracoccidioides*, and *Coccidioides*), and three *Aspergillus* genomes. Using 2,062 single copy core genes present in all strains, we estimated a phylogeny of these organisms using RAxML ([25]; Fig 1). This analysis strongly supports the clustering of *Blastomyces* with *E*. *parva* (100% of bootstrap replicates and 100% Gene Support Frequency (GSF) [26]) as previously reported [19,20]. In contrast to prior work, *Histoplasma* is strongly supported as sister group to *Blastomyces* and *E*. *parva* (100% of bootstrap replicates and 90% GSF), with *E*. *crescens* strongly supported as a sister group to that clade (100% of bootstrap replicates and 100% GSF), and with *Paracoccidioides* in a basal position (Fig 1). The polyphyletic nature of *Emmonsia* suggests that the Ajellomycetaceae have undergone multiple evolutionary transitions allowing the infection of humans and other mammals. Within *Blastomyces*, we found support for strain SLH14081 as an outgroup relative to the other three strains (S3 Fig). This is consistent with the placement of strain SLH14081 within the newly described species *B*. *gilchristii* [3]; the other three strains sequenced here are still classified as *B*. *dermatitidis*.

### *Blastomyces* genomes show a bimodal GC distribution

**Fig 2:**
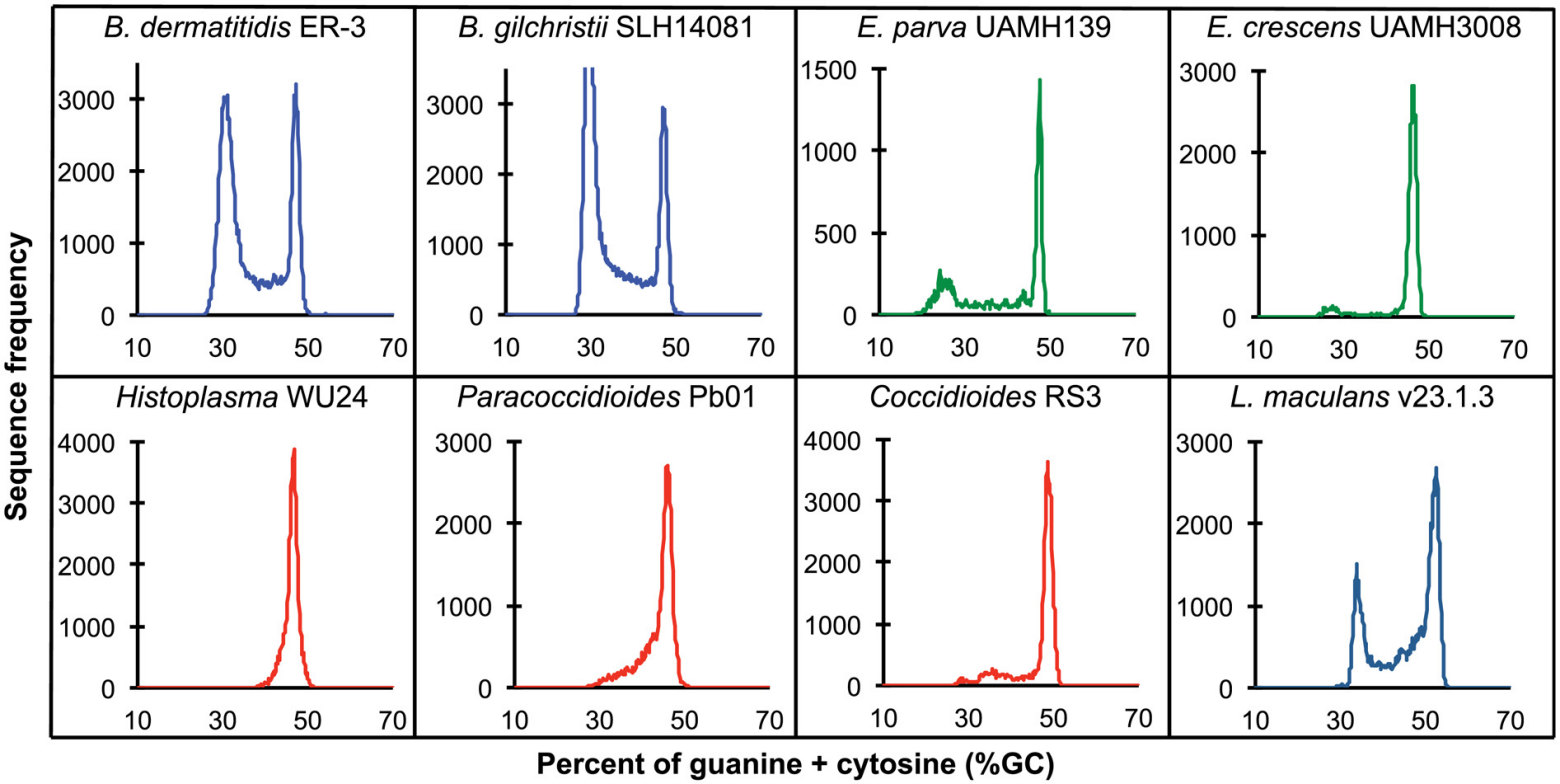
GC frequency distributions (histograms) of overlapping fragments (windows, of 32 kb) of the genome assemblies of *Blastomyces dermatitidis* ER-3, *B*. *gilchristii* SLH14081, *Emmonsia parva* (UAMH139), *E*. *crescens* (UAMH3008), *Histoplasma capsulatum* (WU24), *Paracoccidioides lutzii* (Pb01), *Coccidioides immitis* (RS3), and *Leptosphaeria maculans* (v23.1.3). The bin size of the histograms is approximately 0.1% GC. Horizontal axes show GC % and vertical axes show relative frequencies.

A bimodal distribution of GC-content observed in all *Blastomyces* sequenced, which was less pronounced in *E*. *parva* and *E*. *crescens* and absent in other Ajellomycetaceae, suggests that these genomes are organized in large isochore-like regions of high and low GC-content. This finding for nuclear DNA explains the GC-poor fraction of the *Blastomyces* genome initially identified using CsCl gradient analytical ultracentrifugation [27], which the authors hypothesized was due to a large proportion of GC-poor mitochondrial DNA in *Blastomyces* cells. Examining the genome wide GC content revealed a bimodal distribution for all strains of *Blastomyces* including ER-3 and SLH14081, the smallest and largest assembly, respectively (Fig 2), and was observed for all window sizes ranging from 2 kb to 256 kb (S4 Fig). The detection of a bimodal signal in larger windows supports the organization of the genomes in large isochore-like regions, with average GC content of 29.6% and 31.0% in GC-poor regions and 45.9% and 46.6% for the rest of the genome in *B*. *gilchristii* strain SLH14081 and *B*. *dermatitidis* strain ER-3, respectively (Table 2). Analysis of the related pathogens *H*. *capsulatum*, *P*. *lutzii*, and *C*. *immitis* showed no evidence for bimodality of GC content, while both *E*. *parva* and *E*. *crescens* revealed small peaks of low GC sequence. Read-based analysis and using smaller window sizes (e.g. 128 bp) supported these findings, suggesting they are not due to differences in assembly completeness (S5 Fig).

**Table 2:** Gene and repeat features of *Blastomyces* GC-rich and GC-poor regions compared to *Histoplasma*.

	*Blastomyces*				*Histoplasma*
	GC-poor		GC-rich		
	ER-3	SLH14081	ER-3	SLH14081	WU24
Total size (Mb)	41.1	49.1	25.4	26.2	33
Total genes	1,990	1,858	7,765	7,834	9,251
Gene length (bp)	1,549	1,471	2,737	2,716	1,686
Intergenic distance (bp)	18,523	22,983	1,212	1,400	1,850
GC content (%)	31	29.6	46.6	45.9	46.2
Gene GC content (%)	48	46.9	51.8	51.8	51.2
Coding (%)	7.5	5.6	83.4	80.9	35.9
Syntenic genes (%)	73.4	76.1	99	98.8	NA
Repeat (%)	93.7	95.2	6.2	4.8	15.4

To further examine the organization of GC-content across the genome, we next defined the boundaries of low GC content regions in *Blastomyces*. In the smallest assembly, of the ER-3 strain, we identified 221 GC-poor tracts with an average size of 186.0 kb, encompassing a total size of 41.1 Mb (Tables 2 and S3). In the largest assembly, of the SLH14081 strain, we identified 350 GC-poor tracts with an average size of 140.2 kb, encompassing a total size 49.1 Mb (Tables 2 and S3). The 8 Mb difference between the total size of GC-tracts in the genomes of *B*. *dermatitidis* ER-3 and *B*. *gilchristii* SLH14081 accounts for nearly all of the 8.8 Mb difference in assembly size. Notably, GC-poor tracts in *Blastomyces* can be quite long, and reach maximal lengths of 1.3 Mb. In the assemblies of *E*. *parva*, *E*. *crescens* and other Ajellomycetaceae, long GC-poor tracts were rarely observed (e.g., a total of only 4 GC-poor regions larger than 10 kb in *E*. *parva* were found adjacent to a long GC-rich region in the same scaffold, and just 1 in *E*. *crescens*), corresponding to the less pronounced bimodal GC distribution of the genome assembly. However, more contiguous assemblies would be needed to reveal the overall extent of long GC-poor tracts. The only other fungal genome noted to have an isochore-like structure, *Leptosphaeria maculans* [28], contains a smaller expansion of GC-poor regions (Fig 2); individual tracts were on average half the size (70.4 kb) of those in *Blastomyces*, and encompassed a smaller fraction (36%) of the *L*. *maculans* genome [28]. This difference is consistent with the lower fraction of long AT blocks we observe by comparing windows of different sizes in *Blastomyces* and *L*. *maculans* (S4 Fig).

**Fig 3:**
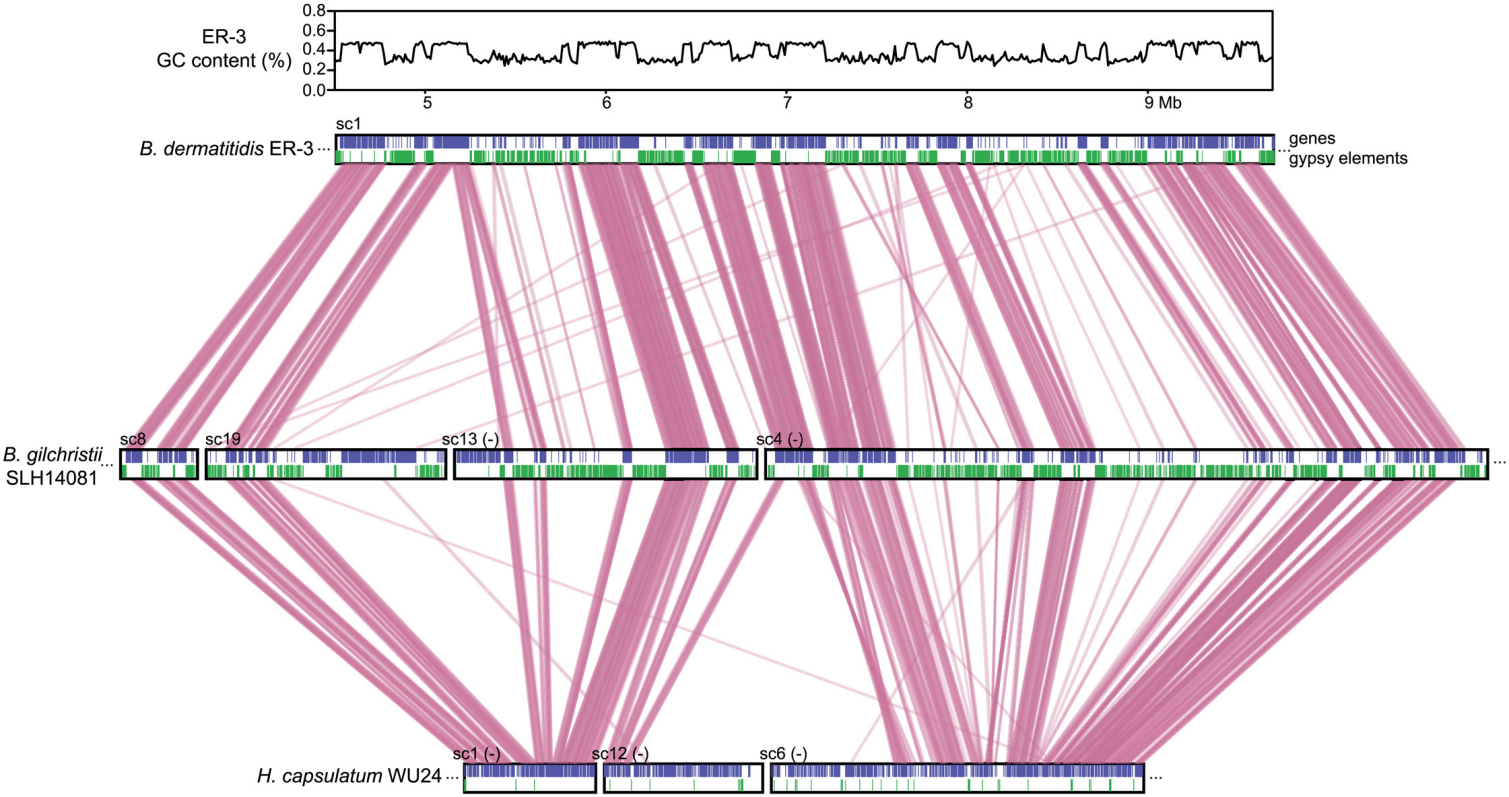
Correspondence of GC content and synteny for *Blastomyces*. Comparison of GC content (top panel) and genome synteny (lower panel) for a 5.2 Mb region of *B*. *dermatitidis* strain ER-3 (scaffold (sc) 1, coordinates from 4.5 to 9.7 Mb) and corresponding syntenic regions of *B*. *gilchristii* strain SLH14081 and *Histoplasma capsulatum* strain WU24. Location of genes (blue boxes) and gypsy elements (green boxes) are depicted across each genomic region. Orthologs between genomes are connected in pink, which are organized into syntenic regions that are disrupted by GC-poor regions in both *Blastomyces* genomes.

The GC-poor regions include nearly all the repetitive elements in the genome and consequently have a lower density of predicted genes (e.g., see Fig 3). In ER-3, 93.7% of repetitive sequence is found in GC-poor regions (Table 2). The gypsy elements that dominate repetitive sequence in the *Blastomyces* genomes have low GC-content; on average those in ER-3 and SLH14081 have respective GC-content of 31.0% and 29.9%, matching the overall GC level of the GC-poor regions (Table 2). GC-poor tracts of *Blastomyces* contain only approximately one fifth of the predicted protein-coding gene set, including some notable genes such as 1,3-beta-glucan synthase component (*FKS1*), *Blastomyces* yeast phase-specific gene (*BYS1*), and one of two *BYS1-*like proteins we identified (S6 Fig and S4 Table). By contrast, *BAD1*, which encodes an essential virulence factor involved in host cell interaction and immune evasion [13], is found within a GC-rich region. Intergenic regions are also larger here than for other genes in the genome; the average intergenic region for ER-3 is 18.5 kb in GC-poor regions, a 3-fold expansion compared to the 6.0 kb genome-wide average (Table 2 and Figs 3 and S6).

The GC-poor regions also show lower synteny between the *Blastomyces* genomes compared to other regions with more typical GC content (e.g., see Fig 3). Overall, *B*. *dermatitidis* strain ER-3 and *B*. *gilchristii* strain SLH14081 shared 125 syntenic blocks including 93.8% and 94.5% of genes, encompassing only 69.5% and 69.3% of each assembly. These percentages are much smaller than those observed among strains of related species (such as 95% and 93% synteny between strains of *P*. *brasiliensis* [29]). The lower synteny among *Blastomyces* strains is largely explained by the proportion of genes found in repeat-rich, GC-poor regions (Table 2 and Fig 3). Nearly all (99%) of genes in GC-rich regions are highly syntenic across *Blastomyces* strains, even between *B*. *dermatitidis* strain ER-3 and *B*. *gilchristii* strain SLH14081. However, the GC-poor regions have more limited synteny even within strains of *Blastomyces* encompassing 74 to 76% of genes in those regions (Table 2 and Fig 3).

Overall, the function, expression, and selective pressure of genes in GC-poor regions appear similar to those genes found elsewhere in the genome. Despite the lower synteny, GC-poor regions are not significantly enriched for *Blastomyces*-specific genes, nor did they show much functional enrichment (S1 Text, S5 Table). Comparing selection pressure on the 7,228 single copy orthologs present in all four *Blastomyces* genomes also did not find a significant difference in the number of genes with high omega values (omega > 1) (Methods). These analyses suggest that despite the dynamic reorganization due to invading gypsy elements, the GC-poor regions do not appear to be fast evolving by these measures. Furthermore, there is no large-scale difference in the expression levels of genes in GC-poor regions. Comparing transcript levels for genes in GC-poor and GC-rich regions, we found that genes in both GC classes show similar expression levels (S7 Fig), again supporting the general similarity of genes found in these two genomic neighborhoods.

### Characterization of repetitive sequence expansion

**Fig 4:**
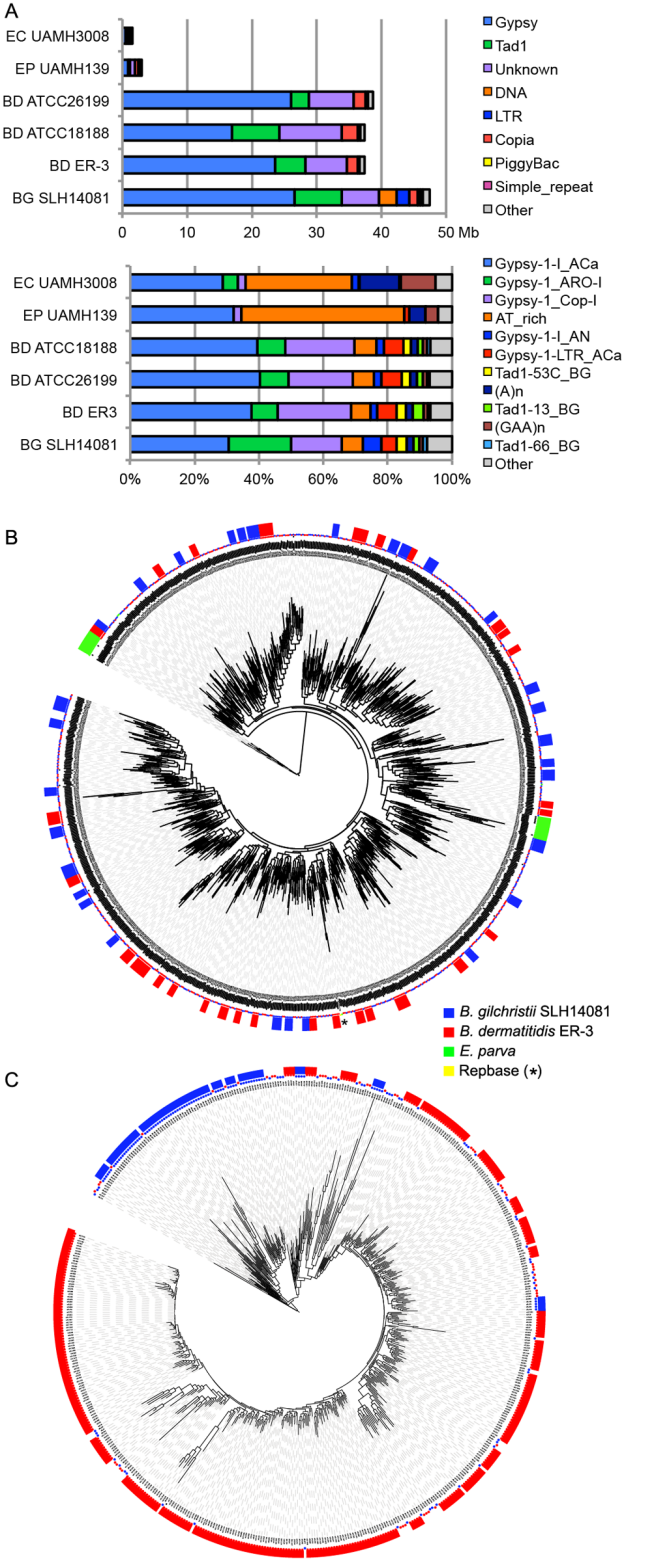
Relative contributions from known repeat categories to *Blastomyces* and *Emmonsia* genomes. **(A)** Repetitive elements were identified in each assembly using a combination of *de novo* classified elements and known elements. The total amount of genome sequence for each element class (top panel) and the relative frequency of known elements (bottom panel) are shown for *B*. *dermatitidis* (BD; ATCC26199, ATCC18188, ER-3), *B*. *gilchristii* (BG; SLH14081), *E*. *crescens* (EC; UAMH3008), and *E*. *parva* (EP; UAMH139). **(B, C)**. Phylogenetic relationship of two subgroups of gypsy elements was inferred using FastTreeDP from alignments of reverse transcriptase domains. The largest subgroup of 922 sequences **(B)** includes domains from the *Blastomyces* strains ER-3 and SLH14081, *E*. *parva* strain UAMH139, and the Repbase ACa Gypsy element, whereas the other subgroup of 544 sequences **(C)** is specific to the two *B*. *dermatitidis* and *B*. *gilchristii*. The outer ring indicates strain specific duplications of four or more sequences.

The 2-fold larger size of the *Blastomyces* genomes compared to other dimorphic fungi is due largely to an expansion of repetitive sequence. The proportions of the *Blastomyces* genome assemblies that were covered by repeats ranged from 56.6% (41.6 Mb) for *B*. *dermatitidis* ATCC18188 to 63.0% (47.5 Mb) for *B*. *gilchristii* SLH14081. SLH14081 had the highest repeat content and the largest assembly size. The *E*. *parva* and *E*. *crescens* assemblies both had a lower repeat content, 9.9% (3.0 Mb) and 5.4% (1.6 Mb), respectively (Table 1). In all genomes, a small number of transposable element classes as well as AT-rich simple sequence regions were highly represented (Fig 4A).

More specifically, the genome expansion in *Blastomyces* strains has resulted from a proliferation of gypsy LTR retrotransposons, including both ancestral and lineage-specific proliferation. In the *Blastomyces* genomes, Gypsy elements account for almost all repetitive DNA, with a lower frequency of sequences similar to the non-LTR Tad1 and copia LTR retroelements (Figs 4A and S8). In all *Blastomyces* and *Emmonsia* genomes the most frequent Gypsy element subtype matches the “ACa” (*A* *jellomyces* or *Histoplasma* *ca* *psulatum*) Gypsy element from Repbase [30] (Fig 4A and 4B). Further phylogenetic characterization of 2,331 Gypsy elements identified four subtypes that appear specific to *Blastomyces* (S1 Text and Figs 4 and S9). Some subtypes had diversities that were primarily the result of ancestral duplication, resulting in large numbers of orthologous elements between strains (e.g., Fig 4B), while other subtypes appeared to predominantly contain strain-specific paralogous expansions, consistent with the cryptic speciation in the *Blastomyces* genus (e.g., Fig 4C). Gypsy elements were also detected in the *Emmonsia* and *Histoplasma* assemblies, but in far fewer copies (Figs 3 and 4), consistent with the recent expansion in *Blastomyces*. Gypsy elements are frequently observed in fungal genomes [31], including *Coccidioides* [32] and *Paracoccidioides* [29] but in far fewer copies.

### Gene family evolution of *Blastomyces* and other Ajellomycetaceae

To identify gene content that could play a role in the evolution of the dimorphism and pathogenesis within the family Ajellomycetaceae, we searched for expansions or contractions in functionally classified genes compared to the other fungi from the order Onygenales (S6 Table). We identified PFAM domains, KEGG pathways, Gene Ontology (GO) terms, or kinase families that were significantly enriched or depleted. Domains associated with polyketide synthases were depleted in the Ajellomycetaceae, and an independent prediction of secondary metabolite enzymes confirmed that *Blastomyces* and other fungi from the Ajellomycetaceae contain fewer PKS gene clusters than other Onygenales (S7 Table, S1 Text). Other differences between the Ajellomycetaceae and other Onygenales include fewer copies of multiple classes of peptidases (M36, M43, S8) as well as an associated inhibitor (I9, inhibitor of S8 protease), variable copy number of LysM-domain proteins potentially involved in chitin binding, which are most expanded in dermatophytes but at next highest levels among the human pathogens in *Blastomyces*, and a higher number of protein kinases (S6 Table and S10 Fig), including an expansion of the FunK1 family similar to that previously noted in *Paracoccidioides* [29].

We next identified 140 gene clusters conserved in *Blastomyces*, *Emmonsia*, *Histoplasma*, and *Paracoccidioides*, but absent from other Onygenales and *Aspergillus* (S8 Table). These gene clusters include a predicted heme oxygenase (BDBG_02689), which could produce free iron from host heme. In addition to the 140 gene clusters, we also identified conserved genes in subsets of the Ajellomycetaceae including homologs of two previously typed antigens; a gene similar to the 27 kDa antigen of *Paracoccidioides* [33] is present in *Blastomyces* and one *Histoplasma* genome, and a gene cluster specific to *Blastomyces* and *Paracoccidioides* shares similarity with the antigenic *Aspergillus* cell wall mannoprotein [34].

### Genes depleted in or absent from *Emmonsia* with possible roles in virulence or phase transitions

To identify potential genetic features of the Ajellomycetaceae pathogenic to immunocompetent humans (*Blastomyces*, *Histoplasma*, and *Paracoccidioides*) relative to *E*. *parva* and *E*. *crescens*, we conducted a second enrichment analysis as described above (S9 Table). The primary pathogens showed enrichment of only two PFAM domains, a phosphorylase and endonuclease (S9 Table). The phosphorylase domain over-represented in *Blastomyces* is associated with nucleoside phosphorylases; many of these proteins also contain Ankyrin repeats and NACHT domains. Phosphorylases are involved in nucleotide and amino acid salvage, and could allow pathogens greater metabolic versatility when certain building blocks are unavailable. The absence of any larger pattern of gain or loss of functional classes suggests that smaller changes in gene content, independent gain and loss between the species, or expression differences may account for differences in pathogenesis.

We then identified specific orthologs present in all four strains of *Blastomyces* but absent from both non-pathogenic *Emmonsia* species. Comparing the ortholog set of *Blastomyces* to *E*. *parva* and *E*. *crescens*, we found a total of 552 ortholog clusters that were present in all *Blastomyces* strains but absent in both *Emmonsia* genomes (S10 Table). Most of these (393 clusters) were present only in *Blastomyces*, and while most of these proteins (92% in *B*. *gilchristii* strain SLH14081) had no PFAM domain assignment, the set did include the *Blastomyces* yeast phase-specific protein 1 (*BYS1*). This gene is a marker of the phase transition to and from the yeast phase [15], although it has recently been shown not to be required for virulence in studied strains [24].

While both *E*. *parva* and *E*. *crescens* are not reported to be primary human pathogens, phylogenetic analysis suggests that the transition to this lifestyle may have been independent, resulting in differential gene loss. One of the genes absent only in *E*. *crescens* is the siderophore iron transporter mirB (*MIRB*). Many pathogenic microorganisms have evolved high affinity iron acquisition mechanisms, which include the production and uptake of siderophores. In *B*. *dermatitidis*, the expression of genes involved in the biosynthesis of siderophores and uptake of siderophores, including two iron transporters (*MIRB* and *MIRC*), are induced under iron-poor conditions [35]. While *MIRB* appears to be absent in *E*. *crescens*, siderophore uptake may be still enabled by the second transporter, *MIRC*, which is conserved in this species.

### Transcriptional profiling of *Blastomyces* in macrophages

**Fig 5:**
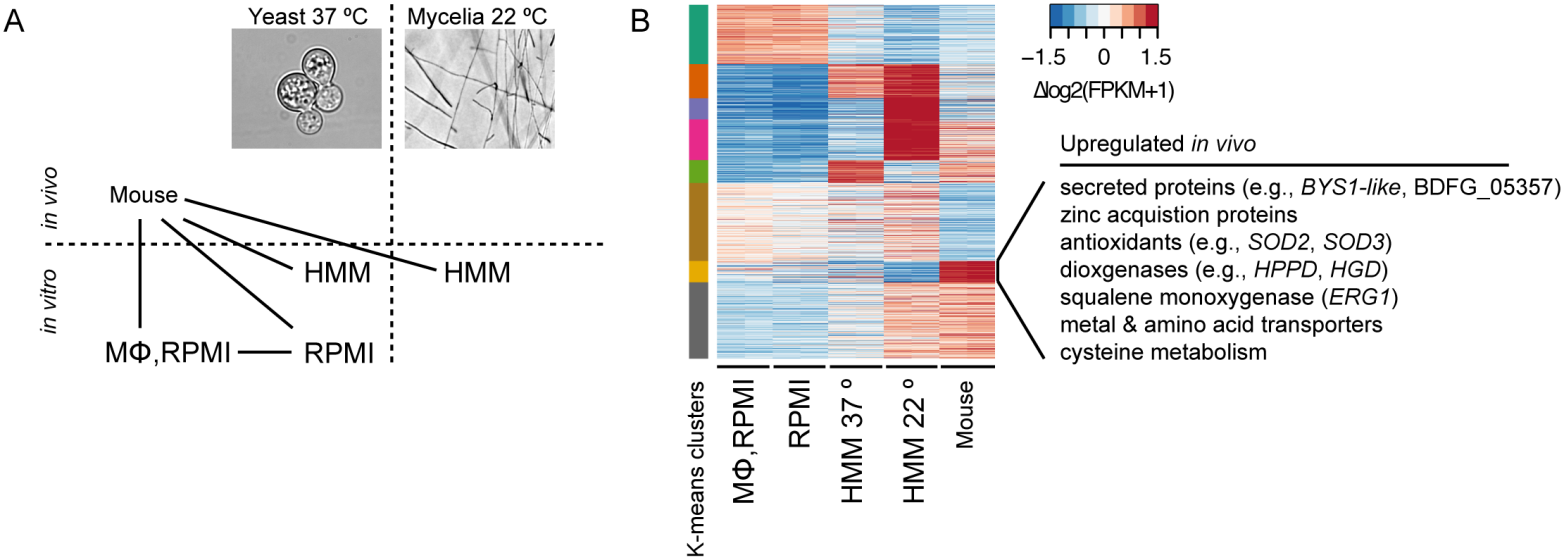
Transcriptional response of *B*. *dermatitidis* strain ATCC26199 to infection. **(A)** Schematic of samples compared by RNA-Seq analysis and **(B)** Heatmap of differentially expressed genes, where the cluster of genes specifically induced *in vivo* during mouse infection is highlighted.

To better understand which *Blastomyces* genes play roles in pathogenicity and virulence, we carried out RNA-Seq of *B*. *dermatitidis* strain ATCC26199 to profile expression under varying temperature, nutrient availability, and infection status. Combining this data allowed us to disambiguate expression variability due solely to differences in temperature and media-specific nutrient availability from those specific to macrophage interactions *in vitro* or host infection *in vivo*. Five conditions were sampled: 37°C with macrophages in RPMI media, 37°C in RPMI media, 37°C in HMM media, 22°C in HMM media, and *in vivo* pulmonary infection with yeast in a mouse model (Fig 5A). For each condition, two biological replicates were performed, and the read counts per transcript were highly correlated between replicates (R> 0.99, S11 Fig). Gene expression levels and mapping statistics are presented in S11 and S12 Tables, respectively.

When *B*. *dermatitidis* yeast cells were co-cultured with human bone marrow derived macrophages, the majority of yeast cells (59%) were internalized by macrophages. Comparison of yeast co-cultured with and without macrophages identified 140 genes differentially expressed between these two conditions, 112 of which were upregulated in the presence of macrophages (S13 Table). This upregulation suggested a small, specific response to macrophages in this experiment. Examination of this set of genes revealed numerous genes that have the potential to facilitate adaptation to the host environment. The 20 most significantly upregulated genes (Table 3) include a predicted secreted endo-1,3(4)-β-glucanase (BDFG_03060) involved in cell separation after cytokinesis in *C*. *albicans* [36], transporters, including an ABC transporter (BDFG_05060) homologous to *Aspergillus fumigatus MDR1* and a zinc transporter (BDFG_02462) similar to the vacuolar zinc transporter *ZRT3 in S*. *cerevisiae*, dehydrogenases involved in amino acid catabolism, and antioxidants peroxisomal catalase (*CATP*, BDFG_02965) and superoxide dismutases (*SOD3*, BDFG_01204; *SOD2*, BDFG_07895), which may protect against reactive oxygen species (ROS). The induction of endo-1,3(4)-β-glucanase and *CATP* in the presence of macrophages was also confirmed by qRT-PCR (S12 Fig and Methods).

**Table 3:** *Blastomyces* transcripts most significantly induced during macrophage infection.

Locus	Predicted function	FDR	Fold Change
BDFG_03193	hypothetical protein, secreted	0	3.63
BDFG_03060	beta-1,3-glucanase,secreted	6.40E-196	2.04
BDFG_06058	transcription factor	6.68E-152	1.90
BDFG_05060	ABC transporter	5.07E-131	1.86
BDFG_04440	transcription factor	2.07E-118	1.54
BDFG_04186	hypothetical protein	1.92E-104	1.58
BDFG_06466	succinate dehydrogenase, iron-sulfur subunit	5.27E-101	1.23
BDFG_06207	vacuolar iron transporter	1.71E-92	1.98
BDFG_04494	succinate dehydrogenase, cytochrome b560 subunit	4.51E-90	1.20
BDFG_01343	electron-transferring-flavoprotein dehydrogenase	5.35E-87	1.17
BDFG_02965	catalase, CATP	4.69E-86	1.30
BDFG_00760	pyruvate decarboxylase	1.26E-84	1.66
BDFG_04269	Eukaryotic cytochrome b561	2.68E-78	1.09
BDFG_04739	hypothetical protein	8.97E-76	3.62
BDFG_02901	cytochrome P450 alkane hydroxylase	1.69E-75	1.05
BDFG_09499	cytochrome c	1.98E-73	1.15
BDFG_04995	hypothetical protein, secreted	3.69E-73	1.54
BDFG_01204	Cu-Zn superoxide dismutase, SOD3, secreted	1.51E-66	1.03
BDFG_02462	Metal ion transporter, ZRT3-like	5.43E-66	1.26
BDFG_04916	hypothetical protein	8.63E-66	1.14

### Transcriptional profiling of *Blastomyces* in a mouse model

We also identified gene expression changes specific to *in vivo* murine pulmonary infection from our transcriptomic data of *B*. *dermatitidis* strain ATCC26199. By k-means clustering of expression values, we identified a set of 72 genes that are upregulated *in vivo* during mouse infection relative to all other conditions, regardless of temperature, media, and *in vitro* macrophage interactions (Fig 5B and S14 Table). Using all conditions for this comparison helped eliminate from consideration differences observed, for example, between the yeast samples cultured in different media. Genes in this set with greater than 2-fold upregulation *in vivo* are highlighted in Table 4, and primarily fell into five functional categories: 1) secreted proteins, 2) zinc acquisition, 3) antioxidants and oxygenases, 4) amino acid metabolism, and 5) transporters.

**Table 4:** *Blastomyces* genes induced during mouse infection*

Locus	Predicted function	Functional categories	Mouse vs Mac	Mouse vs NoMac	Mouse vs Yeast	Mouse vs Mold	Average fold change
BDFG_02039	cysteine synthase	cysteine	0	0	0	0	9.03
BDFG_05357	HRXXH domain protein	secreted, zinc	0	0	0	0	8.11
BDFG_09329	secreted hypothetical protein	secreted	0	0	0	0	7.80
BDFG_02038	MFS transporter	transport	0	0	0	7.28E-304	6.30
BDFG_06873	secreted hypothetical protein	secreted	0	0	0	2.18E-164	5.96
BDFG_09159	zinc transporter, ZRT1	zinc, transport	0	0	3.27E-248	2.57E-312	5.12
BDFG_08433	glycerate kinase		5.28E-208	6.66E-275	1.46E-196	1.30E-83	4.60
BDFG_01204	superoxide dismutase, SOD3	zinc, redox	1.75E-216	0	0	4.67E-223	4.13
BDFG_07895	superoxide dismutase, SOD2	redox	1.99E-168	0		1.82E-316	4.13
BDFG_04319	oxidoreductase	redox	0	0	7.70E-205	1.31E-156	3.66
BDFG_01073	MaoC-like dehydratase		8.90E-77	1.11E-30	0	0	3.34
BDFG_04176	BYS1-like	secreted	6.92E-18	8.39E-53	1.14E-191	0	3.30
BDFG_09115	short chain dehydrogenase	redox	4.76E-90	7.17E-103	3.50E-79	3.98E-126	3.22
BDFG_07137	RNA ligase-like domain protein		8.80E-54	1.60E-44	3.29E-156	1.18E-219	2.82
BDFG_08059	cysteine dioxygenase	redox, cysteine	1.75E-52	5.95E-118	8.41E-294	4.36E-48	2.72
BDFG_05427	cation/proton antiporter	transport	3.58E-82	9.67E-115		0	2.70
BDFG_08334	secreted hypothetical protein	secreted	5.02E-76	1.31E-191	3.21E-31	1.73E-104	2.62
BDFG_00028	2-oxoisovalerate dehydrogenase E1 component, alpha subunit	redox	4.20E-56	2.69E-200	5.14E-285	2.66E-74	2.59
BDFG_02611	acetyl-coenzyme A synthetase		2.18E-108	4.00E-139	4.48E-116	4.64E-120	2.42
BDFG_07269	zinc transporter, ZRT2	zinc, transport	2.20E-162	6.94E-210	9.85E-44	5.74E-117	2.36
BDFG_00760	pyruvate decarboxylase		3.01E-15	1.22E-124	1.11E-272	9.32E-55	2.26
BDFG_05654	2-oxoisovalerate dehydrogenase E2 component	redox	1.63E-35	3.81E-135	7.75E-167	1.18E-87	2.24
BDFG_06615	Sodium:neurotransmitter symporter family	transport	4.85E-62	4.13E-80	3.43E-56	3.56E-119	2.18
BDFG_04184	phenylpyruvate dioxygenase	redox	1.08E-12	9.55E-99	9.00E-211	6.53E-70	2.16
BDFG_00717	CFEM domain protein	secreted	7.48E-15	1.60E-38	1.86E-106	1.27E-32	2.13
BDFG_01386	methionine sulfoxide reductase	redox	2.69E-140	5.79E-137	9.73E-218	2.50E-71	2.13
BDFG_06042	MFS transporter	transport	2.72E-56	2.47E-81	1.90E-31	6.36E-35	2.12
BDFG_03316	MFS transporter	transport	3.98E-44	4.94E-59	7.45E-99	4.48E-84	2.11
BDFG_03902	2-oxoisovalerate dehydrogenase, E1 component, beta subunit	redox	8.15E-37	2.56E-123	2.70E-135	1.48E-105	2.06
BDFG_05401	BTB/POZ-domain protein		9.74E-64	2.92E-88	2.59E-38	9.34E-62	2.02

*Genes with predicted PFAM domains or secretion signals, and greater than 2-fold higher expression during mouse infection are listed; full list of all significant genes in S14 Table.

The most highly expressed gene *in vivo* was *BAD1* (BDFG_03370; S11 Table), which encodes a yeast-phase specific virulence factor that facilitates adhesion to host cells and evasion of host immune defenses [13]. *BAD1* also had the highest transcript abundance for yeast co-cultured with macrophages and yeast without macrophages at 37°C (S13 Table). Thus, *BAD1* was not identified among the set of 72 differentially expressed genes because the transcription of *BAD1* is influenced by temperature [37]. The effect of temperature during the mold to yeast transition on the transcriptome of dimorphic fungal pathogens has been the topic of previous studies [38–41] and was therefore not further evaluated here.

A total of nine secreted proteins were identified in this set of 72, including five of the ten most highly upregulated genes by fold change, potentially playing roles in host-pathogen interactions. Another highly up-regulated secreted protein (BDFG_00717) contains a predicted CFEM domain as well as a GPI-anchor; these features, as well as small size (236 amino acids), are shared with member of the haemoglobin-receptor gene family in *C*. *albicans* [42]. The most highly upregulated gene, BDFG_05357, encodes a HRXXH domain-containing secreted protein that may function as a zinc scavenging protein (Tables 4 and S14). This gene is present in the genomes of *Blastomyces* and *Coccidioides*, but absent from those of *Emmonsia*, *Histoplasma* and *Paracoccidioides*. BDFG_05357 is a homolog of *C*. *albicans PRA1* (pH-regulated antigen-1) [43] and *S*. *cerevisiae ZPS1* (zinc-pH-regulated protein). In *C*. *albicans*, the transcription of *PRA1* and *ZPS1* is induced under alkaline pH and zinc-deplete conditions [44,45], and *PRA1* is co-regulated with its upstream gene, *ZRT1*, which encodes a high-affinity zinc transporter that interacts with zinc-bound *PRA1* [45]. Similarly, the *B*. *dermatitidis* homolog of *ZRT1*, BDFG_09159, is highly expressed *in vivo*; the induced expression of both *PRA1* and *ZRT1* was confirmed by qRT-PCR (S12 Fig). However unlike in *C*. *albicans*, *ZRT1* is not adjacent to *PRA1* in the *B*. *dermatitidis* genome. While *PRA1* is conserved in all four *Blastomyces* genomes, there is no copy of this gene in *Histoplasma* as previously noted [45], nor in *Emmonsia* or *Paracoccidioides*, suggesting differences in how zinc is acquired within the Ajellomycetaceae.

In addition to *PRA1*/*ZPS1* and *ZRT1*, a larger module of genes that regulate zinc acquisition is co-regulated in *Blastomyces*. The transcript abundance of BDFG_07269, which encodes a low-affinity zinc transporter (*ZRT2*), is also significantly upregulated in the mouse model. In *S*. *cerevisiae*, the zinc-responsive transcription factor *ZAP1* regulates expression of *ZRT1* and *ZRT2*, along with *ZPS1*. We identified the ortholog of *ZAP1* in strain ATCC26199 as BDFG_07048, which was also significantly upregulated *in vivo* relative to all other conditions (S14 Table) despite not being identified by k-means clustering. These results suggest that zinc acquisition and homeostasis may play a critical role for survival of *B*. *dermatitidis in vivo*.

Genes that convert reactive oxygen species to dioxygen and dioxygen to metabolites were also highly upregulated *in vivo*. These include two superoxide dismutases (*SOD3*: BDFG_01204 and *SOD2*: BDFG_07895), which were even more upregulated *in vivo* than in macrophages. Four dioxygenases (BDFG_04184, BDFG_04185, BDFG_08059, BDFG_06504) were also upregulated *in vivo*, representing almost half of the dioxygenases found in the genome, which utilize dioxygen to drive amino acid catabolism. This set includes 4-hydroxyphenylpyruvate dioxygenase, (4-HPPD; BDFG_04184) and homogentisate 1,2-dioxygenase (BDFG_04185), which are involved with tyrosine catabolism [46]. Other upregulated oxygenases include indoleamine 2,3-dioxygenase (BDFG_06504) and squalene monooxygenase (*ERG1*—BDFG_07857), which are involved with tryptophan catabolism and ergosterol biosynthesis respectively. *ERG1* is a target of current antifungal drugs, including terbinafine. High *in vivo* expression of this gene may suggest that drugs targeting it may be more effective *in vivo* than *in vitro*.

Genes involved in cysteine biosynthesis and catabolism were highly upregulated during infection including cysteine synthase A (BDFG_02039) and cysteine dioxygenase (BDFG_08059). Cysteine synthase A may provide a large pool of synthesized cysteine for *B*. *dermatitidis* metabolism; the induced expression during infection was confirmed by qRT-PCR (S12 Fig). Based on orthology analysis, cysteine synthase A is absent from the genome of *H*. *capsulatum*, and previous studies have shown that *Histoplasma* yeast are auxotrophic for cysteine [47,48]. Cysteine dioxygenase catabolizes cysteine to L-cysteinesulfinic acid, an intermediate that can be used for taurine biosynthesis or metabolized to sulfite and pyruvate. A homolog of *C*. *albicans SSU1* (BDFG_06814), which encodes a sulfite efflux pump and is co-regulated with cysteine dioxygenase in *C*. *albicans* [49], was also upregulated in *B*. *dermatitidis*.

Transporters in fungi have been associated with an enhanced ability to remove harmful products as well as to survive on diverse nutrient sources, both of which could contribute to virulence and pathogenicity. Of the 72 genes upregulated *in vivo* during mouse infection, 11 are predicted transporters. These included the major facilitator type (MFS; BDFG_06068 –unknown function, BDFG_06042 –glycose transport, BDFG_02038 –unknown function), amino acid transporters (BDFG_02310, BDFG_07447) and metal transporters (zinc/iron transporters discussed above, BDFG_09159, BDFG_07269, and *NIC1* nickel transporter, BDFG_02449; S14 Table). This upregulation potentially reflects differences in metabolite and cofactor availability in the host relative to *in vitro* conditions.

## Discussion

### Phylogenetic position of *Blastomyces* spp. and *Emmonsia parva* and *E*. *crescens*

Our whole-genome based phylogenetic analysis recovered a sister-group relationship between *Blastomyces* spp. and *Emmonsia parva*, as previously reported from ribosomal DNA sequences [19,20]. However, our study placed *Histoplasma* as the next most basal species, and uniquely placed *E*. *crescens* between *Histoplasma* and the basal *Paracoccidioides* with strong bootstrap support. This more external position of *Paracoccidioides* compared to *Histoplasma* agrees with an earlier rDNA tree without *Emmonsia* [50]. Furthermore, gene support frequencies (GSF) were relatively high, and increased when we subsampled only well-supported genes, providing additional support for the topology presented here.

The polyphyletic nature of the non-human pathogen *Emmonsia* suggests substantial plasticity in regard to human pathogenesis in this group. Ancestral variation in the ability of these species to infect other mammals could then be associated with exaptation to human hosts. Additional diversity of *Emmonsia*, including the third described species, *E*. *pasteuriana* [51,52] and other closely related isolates [17] suggests that the full breadth of the *Emmonsia* genus may not be captured by the two isolates sequenced here. Interestingly, both *E*. *pasteuriana* and related isolates produce yeast cells at high temperature, rather than the adiaspores produced by *E*. *parva* and *E*. *crescens*. Further sequencing of *Emmonsia* species and other related strains may reveal additional patterns and trends in the evolution of the dimorphic fungi.

### Genome expansion and segmentation: GC-poor isochore-like regions

The mosaicism observed here in the genome of *Blastomyces* differs substantially from that observed in other fungi and larger eukaryote genomes. While isochore-like GC-poor regions are unprecedented at this scale in fungal genomes described to date, there are parallels to the organization of *L*. *maculans*, though GC-poor regions occupy a smaller fraction of that genome [28]. Longer GC-poor isochores of more than 300 kb are commonly found in mammals and other vertebrates [53–55]. GC-poor isochores in vertebrates are often more stable over long evolutionary times [55,56] and have other properties such as lower gene expression [55] that do not appear to be shared by the GC-poor tracts of *B*. *dermatitidis* and *B*. *gilchristii* (S1 Text).

Characterization of repetitive sequence in GC-poor regions suggests these originated with a dramatic expansion of elements of the LTR/Gypsy category. Phylogenetic analysis suggests these elements swept through a lineage leading to the present-day *B*. *dermatitidis* and *B*. *gilchristii*, and to a lesser extent *Emmonsia parva*, and have further expanded during the diversification of *Blastomyces*. While *H*. *capsulatum* does not have such an expanded genome, or a sizable GC-poor component, and so appears less affected by gypsy expansion, *Histoplasma* may alternatively have more robust defense against repetitive elements or be less able to accommodate large amounts of repeats in its genome.

While GC-poor tracts have been particularly dynamic areas due to Gypsy element insertions during the recent evolution of *Blastomyces*, these regions appear typical in gene content and expression. Perhaps due to their recent origin, the GC-poor regions do not appear to have sequestered particular classes of genes such as secreted proteins or have other hallmarks of rapidly evolving gene content. The long GC-poor regions also include some well characterized genes involved in phase transitions and pathogenesis, including the *Blastomyces* yeast-specific gene *BYS1*, a marker of the phase transition to and from the yeast phase [15,24]. Reduced levels of synteny in the GC poor regions between *B*. *dermatitidis* and *B*. *gilchristii* could prevent effective meiotic recombination between the two lineages, further supporting their designation as separate species.

### Functional diversity of gene content in *Blastomyces* and the other Ajellomycetaceae

Despite the large increase in genome size in *Blastomyces*, the total number of protein coding genes is only modestly expanded. *Blastomyces* and other sequenced species from the Ajellomycetaceae family, including the human primary pathogens *Histoplasma* and *Paracoccidioides*, have similar gene content with only a few gene loss or gain events that map to common functional classes. This stability suggests that more modest differences in gene content and sequence, as well as potential divergence of gene regulation, contribute to phenotypic differences between the species. Larger differences exist between the Ajellomycetaceae and other more divergent members of the Onygenales. There is no expansion of degradative proteases as previously noted for *Coccidioides* [57]; in fact, three peptidase families (M36, M43, and S8) are present at lower copy number in *Blastomyces* and the other Ajellomycetaceae. While *Blastomyces* contains a higher number of LysM proteins than the dimorphic Onygenales, the number is small relative to that found in Dermatophytes [58]. This analysis also identified candidate genes involved in host interaction, including proteins homologous to antigens in related fungi and a heme oxygenase that could release iron from host heme.

### Features of *Blastomyces* gene expression in macrophages and *in vivo*

For yeast co-cultured with macrophages and yeast *in vivo*, some aspects of the transcriptional response were shared including response to oxidative stress and amino acid catabolism. Yeast co-cultured with macrophages showed upregulation of numerous genes involved in oxidative reduction, which may play a major role in protecting *Blastomyces* from ROS. The macrophage phagosome is poor in glucose and amino acids, but rich in ROS [59,60]. *Blastomyces* is relatively resistant to ROS and virulence correlates with the ability to minimize ROS generation in innate immune cells [61,62]. The upregulation of superoxide dismutases (*SOD3*, *SOD2*) and catalase P may protect *B*. *dermatitidis* yeast against oxidative stress. In *H*. *capsulatum*, extracellular *SOD3* and intracellular catalase P, contribute to survival within macrophages [63,64]. Moreover, *SOD3* promotes *H*. *capsulatum* virulence in a murine model of pulmonary infection [63]. The upregulation of 4-HPPD, which is involved with pyomelanin biosynthesis, contributes to antioxidant defense and intracellular survival of *Penicillium marneffei* [65]. Inhibition of 4-HPPD in *P*. *brasiliensis* and *P*. *marneffei* blocks the phase transition to yeast at 37°C [65,66]. Furthermore, *in vivo* numerous dioxygenases were upregulated, suggesting that dioxide produced in response to ROS may be utilized for amino acid metabolism.

Changes in amino acid metabolism were prevalent in both the macrophage co-cultured and *in vivo Blastomyces*, suggesting the recycling of amino acids as an energy source (Results, S1 Text). In particular, the very specific increase in cysteine catabolism (cysteine dioxygenase) and biosynthesis (cysteine synthase A) during *in vivo* infection suggests that cysteine may be critical to virulence. In mice, deletion of cysteine dioxygenase (*CDG1*) in *C*. *albicans* results in attenuated virulence [49]. Furthermore, upregulation of sulfite efflux pump (*SSU1*), which is co-regulated with *CDG1* in *C*. *albicans*, could play a role in *B*. *dermatitidis* virulence during *in vivo* infection. Exported sulfite can destabilize host proteins by reducing disulfide bonds and facilitates the growth of dermatophytes on keratinized tissue [67]. How breakdown of tryptophan by indoleamine 2,3-dioxygenase (IDO), which can supply *de novo* nicotinamide adenine dinucleotide (NAD+), alters the fungal-host interaction is unknown. In cancer, tumor cells with increased expression of IDO may facilitate tryptophan depletion in the microenvironment to suppress the host immune response [68]. Infection with *H*. *capsulatum*, *P*. *brasiliensis*, and *C*. *albicans* upregulates host IDO activity, reduces fungal growth, impairs Th17 T-cell differentiation, and blunts excessive tissue inflammation [69–71].

The specific *in vivo* upregulation of genes that encode secreted proteins as well as those involved with transmembrane transport (e.g., amino acids, glucose), amino acid metabolism (e.g., cysteine), and metal acquisition (e.g., zinc, nickel) highlights virulence factors potentially being missed by *in vitro* studies and the importance of understanding nutrient and co-factor availability in any study system. Uptake of zinc and nickel, which serve as enzyme co-factors, contribute to virulence in *C*. *albicans* and *Cryptococcus neoformans* respectively [45,72]. *PRA1* encodes a secreted “zincophore” under alkaline and zinc-poor conditions that acts in concert with *ZRT1* to promote zinc acquisition and facilitate endothelial cell damage by *C*. *albicans* [45]. NIC1-mediated nickel uptake is critical for urease activity, which contributes to *C*. *neoformans* invasion of the central nervous system [72]. In *C*. *posadasii*, urease induces host tissue damage [73]. While genes involved with the acquisition of zinc (e.g., *ZRT1*, *ZRT2*, *ZAP1* homologs) and nickel (e.g., *NIC1* homolog) are largely conserved with other fungi, the absence of *PRA1* in *Histoplasma*, *Paracoccidioides*, *and Emmonsia* highlights recent evolutionary changes in zinc acquisition mechanisms in the family Ajellomycetaceae. This, in conjunction with differences in cysteine metabolism between *Blastomyces* and *Histoplasma*, suggest that despite the many common elements of dimorphism and pathogenesis, each genus of dimorphic fungi likely has unique nutritional requirements.

## Methods

### Selection of isolates for sequencing

Four strains of *Blastomyces* were sequenced: SLH14081 representing the new species *B*. *gilchristii*, and ER-3, ATCC18188 and ATCC26199 representing *B*. *dermatitidis*. The SLH14081 strain is a highly virulent, clinical isolate that can cause disease in immunocompetent persons, while ER-3 was isolated from a woodpile and is hypovirulent in mice [21,22]. The remaining two strains are strain ATCC18188, a representative MAT 'alpha' isolate, and ATCC26199, a commonly used laboratory isolate.

Two species that are closely related to *Blastomyces*, that can cause pulmonary disease in rodents (adiaspiromycosis), were also sequenced: *Emmonsia parva* UAMH139 and *Emmonsia crescens* UAMH3008. These isolates were chosen for comparison as these species are not typically human pathogens, yet they are closely related to the three pathogenic dimorphic genera *Blastomyces*, *Histoplasma* and *Paracoccidioides*, with which they form a clade that is nested within the order Onygenales and represents the Ajellomycetaceae family [20].

### Sequencing of *Blastomyces*, *E*. *parva* and *E*. *crescens*

Genomic DNA for sequencing was prepared from mycelial or yeast culture, using phenol/chloroform extraction. For the *Blastomyces* SLH14081 and ER-3 strains, whole genome shotgun sequence was obtained using Sanger technology on an ABI 3730 from a Fosmid (epiFOS) and two plasmid (pJAN and pOT) libraries. For *B*. *dermatitidis* ATCC18188, whole genome shotgun sequence was obtained from two small insert libraries (fragment and 1.5 kb) using Roche 454 technology and from a Fosmid library using Sanger technology. For *B*. *dermatitidis* ATCC26199 20X of sequence was generated using 454 technology from a small insert fragment library. In addition, a plasmid (pOT) and Fosmid (epiFOS) library were constructed and sequenced using Sanger technology by the Washington University Genome Center, generating a total of roughly 3.6X coverage.

For each *Emmonsia* species, a single library was used to generate 101 bp paired-end reads using Illumina technology on a Genome Analyzer II generating a total of 116X coverage for *E*. *parva* UAMH139 and 163X coverage for *E*. *crescens* UAMH3009. Libraries of average insert size of 639 bp for *E*. *parva* and of 686 bp for *E*. *crescens* were chosen based on the electropherograms obtained from Bioanalyzer. Sequencing of both *Emmonsia* genomes was performed at the Genomic Sequencing Laboratory, University of California, Berkeley.

### Genome assemblies

*Blastomyces* strains SLH14081 and ER-3 were assembled with Arachne [74] (Assemblez Build 20080911). For *B*. *dermatitidis* ATCC18188, a hybrid assembly was generated with Newbler version 2.3. For *B*. *dermatitidis* ATCC26199, a hybrid assembly of the Sanger and 454 data was generated with Newbler version "MapAsmResearch-03/15/2010" with options-rip and -scaffold.

For the *Emmonsia* genomes, assemblies were generated using multiple programs, including the SOAPdenovo / GapCloser package [75], ABYSS [76] and Velvet [77]. SOAPdenovo assemblies were selected based on quality metrics. While assemblies with high *k* values increased the fraction of GC-poor regions represented in the assembly, optimal assembly of gene sequences were achieved using lower *k* values, based on comparing each assembly to gene sets of *Blastomyces* and other related dimorphic fungi using TBlastN. The assemblies for the *Emmonsia* genomes (*k* = 27 for *E*. *parva* and *k* = 29 for *E*. *crescens*) were processed using the program GAEMR (http://www.broadinstitute.org/software/gaemr/), where overall assembly metrics were used to select the best assembly considering both continuity and completeness, though these measures were lower than for genomes assembled from multiple libraries.

### Optical mapping of *Blastomyces*

To validate the assembly of strain SLH14081 and anchor it onto chromosomes, we constructed an optical map, a single-molecule based ordered restriction map. The map of *B*. *gilchristii* strain SLH14081 was constructed by OpGen using the restriction enzyme BsiWI (C^GTACG). The optical map consists of 16 linkage groups, with size ranging from 9.728 Mb to 730 kb. The total size of the map was estimated as 79.64 Mb in size, slightly larger than the 75.35 Mb genome assembly, likely due to repetitive element sequence missing from the assembly. A total of 36 assembly scaffolds covering 68.9 Mb were mapped based on shared restriction sites to the optical linkage groups (S2 Table).

### RNA-Seq of ATCC26199 from yeast, mold, and infection conditions

To enable more accurate gene prediction and analyze gene expression, RNA was prepared and deeply sequenced from five conditions (yeast with or without macrophages in RPMI media, *in vivo* during murine pulmonary infection, and *in vitro* yeast and mold in *Histoplasma* macrophage media (HMM)) with two biological replicates per condition.

ATCC26199 yeast cells were co-cultured with bone marrow derived murine macrophages from C57BL/6 mice in RPMI media with 10% heat inactivated FBS and supplemented with penicillin (100 U) and streptomycin (100 ug) or incubated in this media alone. Yeast and macrophages were co-cultured using a ratio of one yeast for every two macrophages (MOI 0.5). The use of alveolar macrophages was precluded due to the large numbers of mice that would be needed to harvest these cells. Following inoculation of cell culture flasks with *B*. *dermatitidis* yeast, the co-cultures were incubated at 37°C for 24 hrs. The majority of the yeast were either single cells or cells with one bud (average 89%). The extent of macrophage internalization of yeast was measured using Uvitex staining to differentiate between extracellular and intracellular yeast. A total of 1,976 cells were counted across seven individual fields of view, with an average of 59% Uvitex negative (intracellular) and 41% Uvitex positive (extracellular). The majority of *B*. *dermatitidis* cells exhibited yeast morphology (> 96%); pseudohyphal growth occurred in 2.4% of co-cultured yeast and 3.7% of yeast grown in RPMI media without macrophages. Harvested yeast cells were flash frozen in liquid nitrogen, ground with a mortar and pestle into a fine powder, and RNA extracted using the phenol-guanidium thiocyanate-1-bromo-3-chloropropane extraction method [78].

For *in vivo* transcriptional profiling, C57BL/6 mice were infected with 2 x 10^3^ *B*. *dermatitidis* ATCC26199 yeast cells intratracheally and monitored for signs and symptoms of infection [79]. Mice with euthanized by carbon dioxide at 17 days post infection and yeast were isolated from murine lung tissue using the technique developed by Marty et al. [80]. Briefly, excised lungs were homogenized in pre-chilled (4°C) double-distilled, sterile water (ddH_2_O) supplemented with DNase I 10 μg/ml (Roche) using an Omni TH tissue homogenizer (Omni International, Kennesaw, GA), passed through a 40 μm cell strainer (ThermoFisher Scientific, Waltham, MA), and centrifuged at 770g for 5 minutes at 4°C. The supernatant and interface were removed using a serologic pipette and yeast pellet was washed with ice-cold ddH_2_O and rapidly frozen in liquid nitrogen for RNA extraction. Time *ex vivo* was less than 30 minutes and samples were near-freezing (4°C) during all isolation steps. Quality control analyses using qRT-PCR demonstrated that the short *ex vivo* time (< 30 minutes) at 4°C minimized changes in transcript abundance that would have occurred if the samples were processed at higher temperatures or for a longer duration [80]. Total RNA isolated from *B*. *dermatitidis* yeast during pulmonary infection was divided into 2 pools of 5 mice each (pool #1 and pool #2).

*In vitro* yeast were incubated in liquid *Histoplasma* macrophage media (HMM) at 37°C while shaking [81]. The majority of cells had yeast morphology; less than 3.25% of cells grew as pseudohyphae. To generate mycelia, yeast cells were incubated in liquid HMM for 14 days at 22°C while shaking. Harvested yeast and mycelial cells were flash frozen in liquid nitrogen, ground with a mortar and pestle into a fine powder, and RNA extracted using the phenol-guanidium thiocyanate-1-bromo-3-chloropropane extraction method [78].

Total *B*. *dermatitidis* RNA from all samples (*in vivo*, *in vitro*, co-cultures) was treated with TurboDNase (Bio-Rad, Hurcules, CA) and cleaned using an RNeasy column (Qiagen). RNA integrity and quality was assessed using Nanodrop spectrophotometry, 0.8% agarose gel electrophoresis, and an Agilent Bioanalyzer (Agilent Technologies, Santa Clara, CA). RNA integrity numbers (RIN) for *in vivo* samples were > 7.5 (7.6 for pool #1, 7.8 for pool #2). RIN values for *in vitro* and co-cultures (including yeast only RPMI) were ≥ 8.7.

For RNA-Seq, poly-A mRNA was purified for each total RNA sample and strand-specific libraries prepared as previously described [82,83]; each library was sequenced using Illumina Technology, generating an average of 65,174,908 101 bp reads per sample. RNA-Seq was incorporated into gene prediction and used to detect differentially expressed transcripts as described below.

### Genome annotation

For initial gene sets, a total of 38,405 ESTs generated from yeast and mycelial samples of ATCC26199 (Washington University) and from a normalized cDNA library of SLH14081 (Broad Institute) were used for gene prediction. To achieve better transcript coverage, strand-specific RNA-Seq data from 10 samples representing the above yeast, mold, and infection stages was assembled with the Inchworm component of Trinity [84] and processed with PASA [85] to generate a set of transcripts for gene prediction. Gene sets were generated by using EvidenceModeler (EVM) [85] to select the best gene call for a given locus from the gene prediction programs SNAP, Augustus, Geneid, and Genewise and from PASA RNA-Seq transcripts as previously described [85,86].

Project numbers and locus tag prefixes assigned to gene sets are as follows: *B*. *gilchristii* SLH14081 (PRJNA41099, locus tag prefix BDBG), *B*. *dermatitidis* ER-3 (PRJNA29171, prefix BDCG), ATCC18188 (PRJNA39265, prefix BDDG), and ATCC26199 (PRJNA39263, prefix BDFG); the *E*. *parva* UAMH139 (PRJNA178178, prefix EMPG) and *E*. *crescens* UAMH3008 (PRJNA178252, EMCG).

### Expression profiling

RNA-Seq reads were aligned to the transcript sequences of *B*. *dermatitidis* strain ATCC26199 using Bowtie [87]. Transcript abundance was estimated using RSEM [88], TMM-normalized FPKM for each transcript were calculated, and differentially expressed transcripts were identified using edgeR [89], all as implemented in the Trinity package version r2013-2-25 [90]. To identify GO term enrichment of differentially expressed genes, we classified transcripts using Blast2GO [91] and then performed comparisons with Fisher’s exact test. A 2-fold difference in FPKM values and a false discovery rate below 0.05 were used as a criteria for significant differential expression. To identify possible functions of the gene products of significantly differentially expressed parasitic-phase genes, protein homologs were assigned based on BLAST, Gene Ontology (GO) terms and protein family domains (PFAM, TIGRFAM).

### Quantitative real-time PCR (qRT-PCR)

Total RNA was extracted from *B*. *dermatitidis* yeast as described above. One microgram of DNase-treated total RNA was converted to cDNA using iScript cDNA synthesis kit (Bio-Rad). qRT-PCR was performed with SsOFast EvaGreen Supermix (Bio-Rad) using a MyiQ real-time PCR detection system (Bio-Rad). Reactions were performed in triplicate using the following conditions: 1 cycle 95°C x 30 sec, followed by 40 cycles at 95°C for 5 sec, 60°C for 10 sec. Transcript abundance for genes of interest were normalized relative to the transcript abundance of GAPDH. Relative expression (RE) was calculated as RE = 2^-ΔCt^, ΔCt = Ct_gene of interest_−Ct_GAPDH_ [92].

Primer sequences used were as follows: AATCCTTTGACAGTGAAAC (forward) and CCATAAATCTGCTACAACAG (reverse) for BDFG_03060, ACTGTCGGTGGAGAGAAG (forward) and ACTGGGGTGTTGTTGAAG (reverse) for BDFG 02965, GACTATCCCATCCACAAC (forward) and TACAGAGCGGAATCTTTG (reverse) for BDFG 05357, TTTGGCACTGGAGTTATG (forward) and TGCTTCGTAGTCTAAAGTC (reverse) for BDFG 09159, GTGCTACAACGGAGATAC (forward) and GATAACCACCACGAACAC (reverse) for BDFG 02039, ACCCCCGCTCCTCCATCTTC (forward) and GAGTAGCCCCACTCGTTGTCATACC (reverse) for BDBG_07959 (GAPDH).

### Segmentation and identification of genes and repeats located in GC-poor tracts

We used the IsoFinder GC segmentation program (http://bioinfo2.ugr.es/oliver/isofinder; [93]) to segment all ER-3 and SLH14081 scaffolds into long homogeneous genomic regions (LHGRs). The option p2 (parametric/student *t*-test with different variances), a window size of 5 kb and a *p* value cutoff of 0.01 (*P* parameter 0.99) were chosen after evaluating *P* cutoffs between 0.95 and 0.99, and window sizes ranging between 3 and 5 kb. The final settings were chosen as they accommodated gene synteny between ER-3 and SLH14081 in the GC-poor segments, obviating the need to manually remove narrow GC peaks caused by short genic regions.

To identify the coordinates of the longer GC-poor and GC-rich tracts on the assemblies of *Blastomyces* strains ER-3 and SLH14081, we set the boundary between GC-poor and GC-rich at 38% GC, a value that is in the deep valley between the two components of these genomes’ bimodal GC distribution. The deep valley is robust and persists over a wide range of window/segment sizes ranging up to > 60 kb (S4 Fig). We then grouped adjacent segments located between successive transitions (regime switches) across the 38% GC border. Islands of N’s in the interior of the GC-poor tracts were retained, but those at the tract fringes (i.e., next to a jump across the 38% GC threshold) were not. This procedure yields a large-scale segmentation of all scaffolds into strictly alternating “GC-poor” and “GC-rich” tracts. The GC-poor tracts and genes in those regions are listed in S3 and S4 Tables, respectively; GC-rich tracts form the remainder of the assemblies. We performed MySQL joins to identify the genes and repeats (GFF files produced by RepeatMasker of elements from RepeatModeler) located entirely or partly in the GC-poor tracts.

### Synteny analyses

DAGchainer [94] was used to identify syntenic blocks with a minimum of 6 genes, which were required to be in the same order and orientations in the compared genomes. Synteny plots were generated using a custom perl script, using the GDgraph library; code is available at https://github.com/gustavo11/syntenia. Geneious Pro was used to visualize smaller-scale syntenies within and among genome assemblies.

### Recognition and characterization of repeats

*De novo* repetitive sequence in each assembly was identified using RepeatModeler version open-1.0.7 (www.repeatmasker.org/RepeatModeler.html). Copies of *de novo* repeats and fungal sequences from RepBase [95] were mapped in each assembly using RepeatMasker version open-3.2.8 (www.repeatmasker.org/). For phylogenetic analysis of gypsy elements, reverse transcriptase domains were identified from each element; matches to the PFAM RVT_1 domain were identified with HMMER (version 3.1b1) [96] for 6-frame translations of each element generated by EMBOSS transeq (version 6.5.7 with parameters-frame 6-clean Y) [97]. The best domain match for each element was selected, requiring 50% alignment coverage and c-Evalue < 1e-5. The domains identified in *Blastomyces* SLH14081 (991 total) and ER-3 (1,296 total), *E*. *parva* (40 total), and similar Repbase gypsy elements (4 total) were aligned with MAFFT (version 6.717) [98], and a phylogeny estimated using FastTreeDP (version 2.1.8) [99]. Four large subgroups were identified and visualized using iTOL [100].

### Identification and analysis of orthologs and phylogenetic analysis

A total of 16 genomes from the Onygenales order and three *Aspergillus* genomes were chosen for comparative analyses (S15 Table). These include the four *Blastomyces* (SLH14081, ATCC26199, ATCC18188, ER-3) and two *Emmonsia* species (UAMH139, UAMH3008) as well as the following: *Histoplasma capsulatum* WU24 (AAJI01000000), *H*. *capsulatum* G186AR (ABBS01000000), *Paracoccidioides lutzii* Pb01 (ABKH02000000), *P*. *brasiliensis* Pb03 (ABHV02000000), and *P*. *brasiliensis* Pb18 (ABKI02000000), *Coccidioides immitis* RS (AAEC00000000), *C*. *posadasii* C735 delta SOWgp (ACFW00000000), *Uncinocarpus reesii* 1704 (AAIW00000000), *Microsporum gypseum* CBS118893 (ABQE00000000), *Trichophyton rubrum* CBS118892 (ACPH01000000), *Aspergillus nidulans* FGSC A4 (AACD00000000), *A*. *flavus* NRRL3357 (AAIH00000000), *A*. *fumigatus* Af293 (AAHF01000000). OrthoMCL was used to cluster the protein-coding genes of the 19 chosen genomes by similarity.

To estimate the species phylogeny, a total of 2,062 orthologs present in a single copy in all of the 19 genomes were identified. Protein sequences of the 2,062 genes were aligned using MUSCLE, and a phylogeny was estimated from the concatenated alignments using RAxML v7.7.8 with model PROTCATWAG. To more closely examine the relationship of the *Blastomyces* isolates, single copy orthologs were identified in all four strains of *Blastomyces* and *E*. *parva*; the protein sequences of a total of 6,605 single copy orthologs were aligned using MUSCLE, and the resulting sequences replaced with the corresponding codons. A phylogeny was estimated from this nucleotide alignment using RAxML v7.3.3 with model GTRCAT. A total of 1,000 bootstrap replicates were used for each analysis. The level of support for the best RAxML tree was also evaluated using individual gene trees, by calculating the gene support frequency (GSF, [26]). A phylogeny was estimated and bootstrapped using the same parameters as for the concatenated sequence matrix, and gene trees with high bootstrap support at all nodes were then selected. A total of 162 gene trees were supported by at least 70% of bootstrap replicates at all nodes; the percent of gene trees supporting the RAxML best tree was calculated using RAxML and is shown in Fig 1. We also evaluated larger subsets of trees including those with 60% bootstrap support at all nodes, 50% bootstrap support, or all trees regardless of support, and found lower support respectively in each subset for our best tree.

To examine selective pressure on genes in GC-poor regions, we identified 7228 genes that were single copy in the four *Blastomyces* genomes from the OrthoMCL run. d_N_/d_S_ values for each gene were computed on codon-based nucleotide alignments with the codeml module of PAML [101], using the one-ratio (M0) model.

### Gene family and protein domain analysis

Genes were functionally annotated by assigning PFAM domains, GO terms, and KEGG classification. HMMER3 [96] was used to identify PFAM domains using release 27. GO terms were assigned using Blast2GO [91], with a minimum e-value of 1x10^-10^. Protein kinases were identified using Kinannote [102] and divergent FunK1 kinases were further identified using HMMER3. Secondary metabolite gene clusters were predicted with antiSMASH version 2.0.2 [103]. Genes were clustered using OrthoMCL [104] with a Markov inflation index of 1.5 and a maximum e-value of 1x10^-5^.

To identify functional enrichments in *Blastomyces* and other subsets of the 19 compared genomes, we used four gene classifications: OrthoMCL similarity clusters (see above), PFAM domains, KEGG pathways, and Gene Ontology (GO), including different hierarchy levels. A gene was considered to be a member of a given gene class when, respectively, the gene (a) belonged to the given OrthoMCL cluster, (b) contained at least one instance of the given PFAM domain in the encoded protein, (c) belonged to the given KEGG pathway, or (d) was tagged by the given GO label. Using a matrix of gene class counts for each classification type, we identified enrichment comparing two subsets of queried genomes using Fisher’s exact test. Fisher’s exact test was used to detect enrichment of PFAM, KEGG, or GO terms between groups of interest, and p-values were corrected for multiple comparisons [105]. Significant (corrected p-value < 0.05) PFAM and GO terms expansion or depletion was examined for three comparisons: Ajellomycetaceae compared to other Onygenales (S6 Table), pathogenic compared to non-pathogenic from Ajellomycetaceae (S9 Table), and *Blastomyces* compared to other Ajellomycetaceae; the only terms found to be expanded only in *Blastomyces* included nucleosome and zinc ion binding. No significant enrichment in KEGG terms was detected for these comparisons.

## Supporting Information

S1 Fig Optical map of *Blastomyces gilchristii* strain SLH14081. (PNG)

S2 Fig Conservation of core eukaryotic gene (CEG) set across *Blastomyces*, *Emmonsia*, and other compared genomes. The percent coverage of genes with significant Blast similarity is shown for alignments above and below the recommended 70% coverage threshold; matches with less than 70% coverage suggest these are partial gene structures.
(PDF)

S3 Fig Phylogeny of *Blastomyces* and *Emmonsia parva*. Maximum likelihood tree of the four *Blastomyces* strains (ATCC26199, ATCC18188, ER-3, SLH14081) and *E*. *parva* (UAMH139) was inferred using RAxML based on the concatenated nucleotide sequence alignment 6,605 genes. (PDF)

S4 Fig GC frequency distributions (histograms) of overlapping fragments (windows, subsequences) of the genome assembly of *Blastomyces gilchristii* (SLH14081) *B*. *dermatitidis* (ER-3) and *Leptosphaeria maculans* (v23.1.3). Window sizes included 2, 8, 32, 64, 128 and 256 kb. Step size was 1/128 of the window size. The bin size of the histograms is approximately 0.1% GC. Horizontal axes show GC percent and vertical axes show relative frequencies.
(PDF)

S5 Fig GC frequency distributions (histograms) of small overlapping fragments (windows, of 128 bp) of the genome assembly of *Blastomyces dermatitidis* (ER-3), *B*. *gilchristii* (SLH14081), *Emmonsia parva* (UAMH139), *E*. *crescens* (UAMH3008), *Histoplasma capsulatum* (WU24), *Paracoccidioides lutzii* (Pb01), *Coccidioides immitis* (RS3) and *Leptosphaeria maculans* (v23.1.3). (PDF)

S6 Fig Comparison of GC-poor insertions in an otherwise syntenic region of *Emmonsia parva* (UAHM139) and the four sequenced strains of *Blastomyces*. The example illustrates the intraspecific variability in presence/absence of GC-poor segments or ‘inserts’ and, even where their presence and location are conserved, the variability in their lengths. In **(A)** the dotplot of one complete scaffold of *E*. *parva* aligned to *B*. *gilchristii* strain SLH14081 (top) and *B*. *dermatitidis* strain ER-3 (bottom). In **(B)** the corresponding location of the inserts and the length; only insertion sites that were >15 kb for at least one strain are shown. This 265 kb region of the *E*. *parva* genome, lacks intermediate-sized (>15 kb) or long inserts, allowing its use as a simple reference for marking positions.
(PDF)

S7 Fig Comparison of the expression of GC-poor genes vs. GC-rich genes. **(A)** Box plot of the gene expression (log2(FPKM+1)) of the genes located in GC-rich regions (blue) and genes located in GC-poor regions (green) in all five conditions of the RNA-Seq experiment of *B*. *dermatitidis* strain ATCC26199. Histograms in **(B)** show in the *x*-axis the distribution of the gene expression (log2(FPKM+1)) of those genes according their location during mouse infection. Similar distribution was observed in the other four conditions.
(PDF)

S8 Fig Distribution of known repeats families in GC-poor regions as compared with known repeats families in GC-rich regions in *Blastomyces* (ER-3). The list in the left box represent the first 20 LTR/Gypsy representing approximately 90% of the LTR/Gypsy family in the GC-poor regions.
(PDF)

S9 Fig Phylogenetic characterization of Gypsy elements in *Blastomyces*. Four divergent clades of gypsy elements (**A**, **B**, Fig 2B and 2C) were identified from a phylogeny inferred using FastTreeDP from alignments of reverse transcriptase domains identified in gypsy elements of *B*. *dermatitidis* (ER-3), *B*. *gilchristii* (SLH14081) and *E*. *parva* (UAMH139). Each of the four clades is shown separately; **A**. Subgroup of 220 sequences includes non-ACa Repbase elements. **B**. Subgroup of 554 sequences specific to *Blastomyces*. The outer circle indicates strain specific duplications of four or more sequences.
(PDF)

S10 Fig Eukaryotic protein kinase superfamily members (kinomes). The kinomes of *Blastomyces gilchristii* (Bg; SLH14081) and *B*. *dermatitidis* (Bd; ER-3, ATCC26199 and ATCC18188) were compared with *Emmonsia parva* (Ep; UAMH139*)*, *E*. *crescens* (Ec; UAMH3008), *Paracoccidioides brasiliensis* (Pb; Pb18) and *Coccidioides immitis* (Ci; RS3). Kinases are classified into major groups shown as colored blocks. Abbreviations: AGC, protein kinases A; CAMK, calcium/calmodulin-dependent kinases; CK1, casein kinase 1; CMGC, cyclin-dependent kinases (CDK), mitogen-activated, glycogen-synthase, and CDK-like kinases; STE, sterile phenotype kinases; FunK1, fungal-specific kinase 1; PKL, protein kinase subdomain-containing proteins; STK, serine/threonine protein kinase; STE, sterile phenotype kinases; TKL, tyrosine kinases.
(PDF)

S11 Fig Correlation coefficients of FPKM values between samples. Two biological replicates for each condition of the RNA-Seq of *Blastomyces dermatitidis* (ATCC26199).
(PDF)

S12 Fig Quantitative real-time PCR (qRT-PCR) analysis. **(A)** qRT-PCR analysis of endo-1,3(4)-β-glucanase (BDFG_03060) and catalase P (*CATP*; BDFG_02965) from *B*. *dermatitidis* ATCC26199 yeast cells co-cultured with macrophages (Macrophage) and yeast cells grown in the absence of macrophages (No Macrophage) at 37°C in RPMI. **(B)** qRT-PCR analysis of genes encoding a zinc-scavenging protein (*PRA1*; BDFG_05357), zinc transporter (*ZRT1*; BDFG_09159), and cysteine synthase A (*CSA*; BDFG_02039) from *B*. *dermatitidis* ATCC26199 yeast cells isolated during murine pulmonary infection (*in vivo*) and yeast cells co-cultured with macrophages (Macrophage) in RPMI at 37°C. qRT-PCR data are from 2 experiments. Relative expression (RE) for the target gene was compared to GAPDH: RE = 2^-ΔCt^ = 2^-(gene of interest)–(GAPDH)^.
(EPS)

S1 Table Phenotypic differences observed among *B*. *dermatitidis*, *E*. *parva* and *E*. *crescens*. (DOCX)

S2 Table Optical map information for *B*. *gilchristii* strain SLH14081. (DOCX)

S3 Table Coordinates of GC-poor tracts in *B*. *dermatitidis* ER-3 and *B*. *gilchristii* SLH14081. (XLSX)

S4 Table Genes in GC-poor tracts in *B*. *dermatitidis* ER-3 and *B*. *gilchristii* SLH14081. (XLSX)

S5 Table Significant PFAM and GO enrichments comparing genes in GC-poor regions. (XLSX)

S6 Table Significant gene class enrichments in Ajellomycetaceae compared to other Onygenales. (XLSX)

S7 Table Secondary metabolite gene clusters. (DOCX)

S8 Table Gene clusters conserved in *Blastomyces*, *Emmonsia*, *Histoplasma*, and *Paracoccidioides*. (XLSX)

S9 Table Significant gene class in human pathogenic from Ajellomycetaceae (Blastomyces/*Paracoccidioides*/*Histoplasma* vs *E*. *crescens*/*E*. *parva*). (XLSX)

S10 Table *Blastomyces* genes absent in *E*. *parva* and *E*. *crescens*. (XLSX)

S11 Table Gene expression levels. (XLSX)

S12 Table RNA-Seq mapping statistics. (DOCX)

S13 Table List of significantly upregulated genes in yeast-Macrophages as compared with yeast-RPMI. (XLSX)

S14 Table List of significantly upregulated genes *in vivo*. (XLSX)

S15 Table List of genomes of Onygenales and *Aspergillus* species compared in this study. (DOCX)

S1 Text Supplementary Notes. Possible biological meaning of the GC-poor tracts;
Functional enrichment of genes in GC-poor tracts; The GATA transcription factor *SREB* and siderophore use; Secondary metabolite biosynthesis clusters; Characterization of gypsy element expansion; Gene expression changes in amino acid metabolism.
(DOCX)
