# Supplementary figures and images for: The Dynamic Genome and Transcriptome of the Human Fungal Pathogen Blastomyces and Close Relative Emmonsia
Source: PLoS Genet. 2015 Oct 6;11(10):e1005493. doi: 10.1371/journal.pgen.1005493 (PMC4595289; doi:10.1371/journal.pgen.1005493)

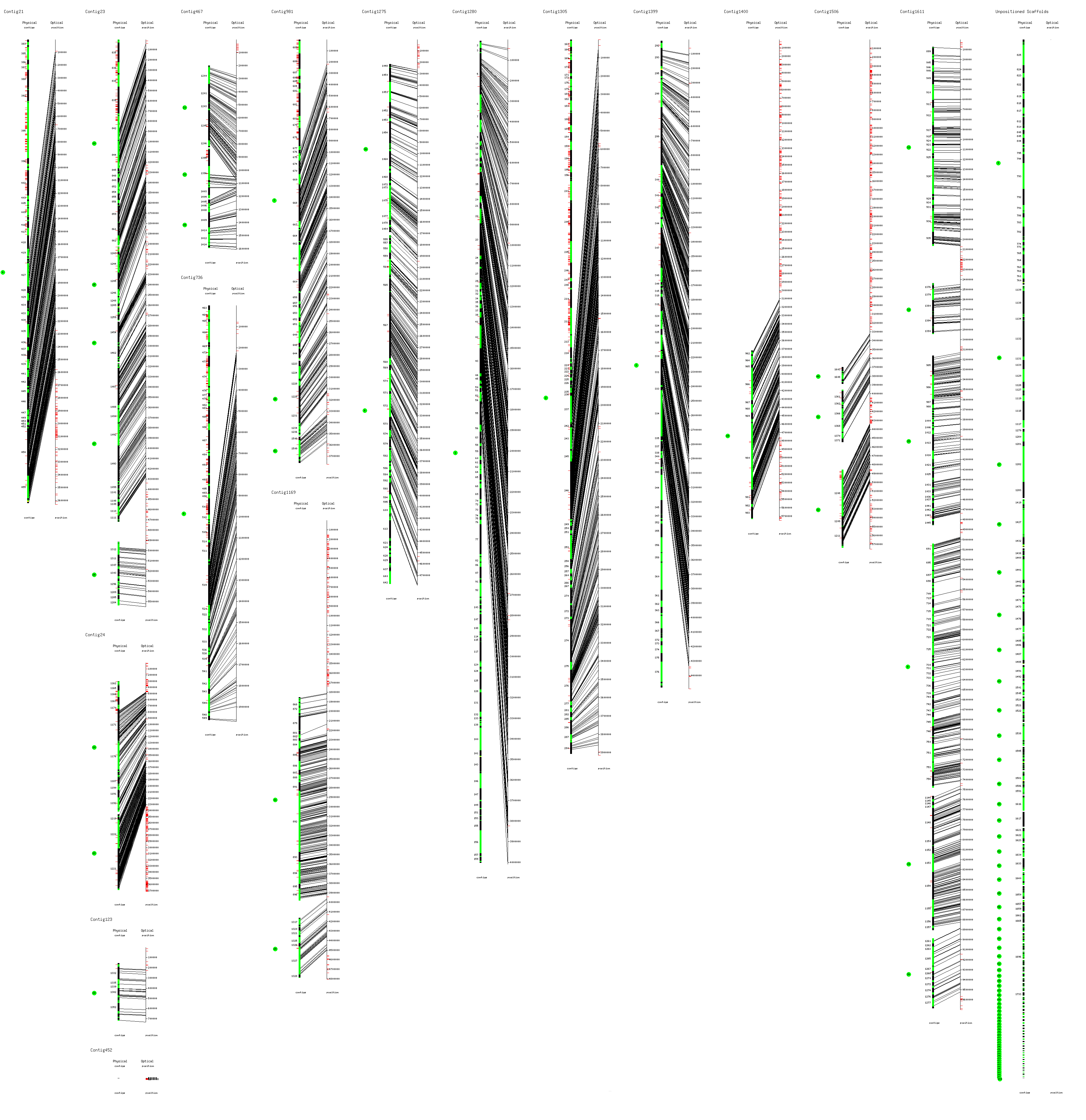

Supplement: S1 Fig — (PNG) [file pgen.1005493.s001.png]

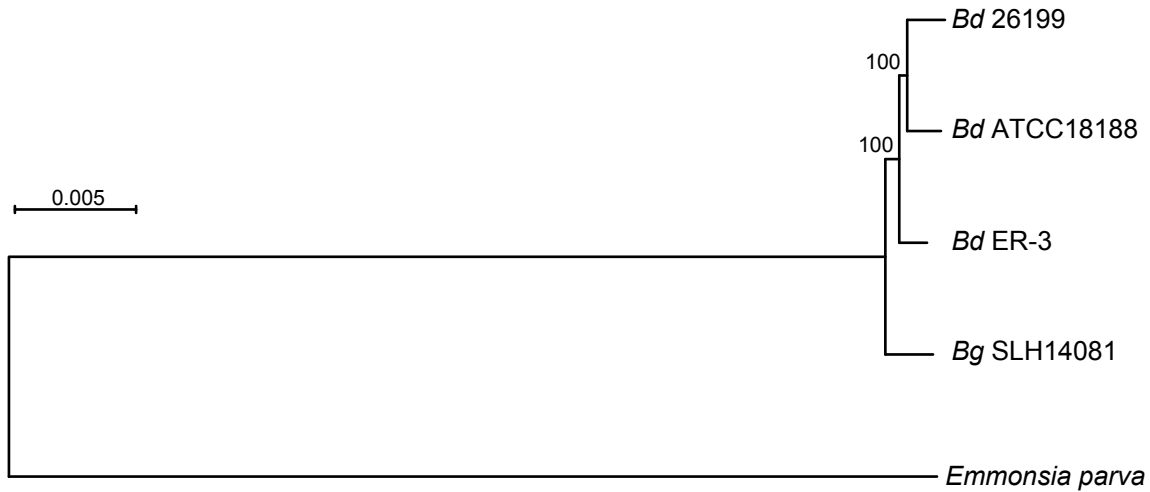

Supplement: S3 Fig — (PDF) [file pgen.1005493.s003.pdf]

***Bg* SLH14081**

**Sequence frequency**

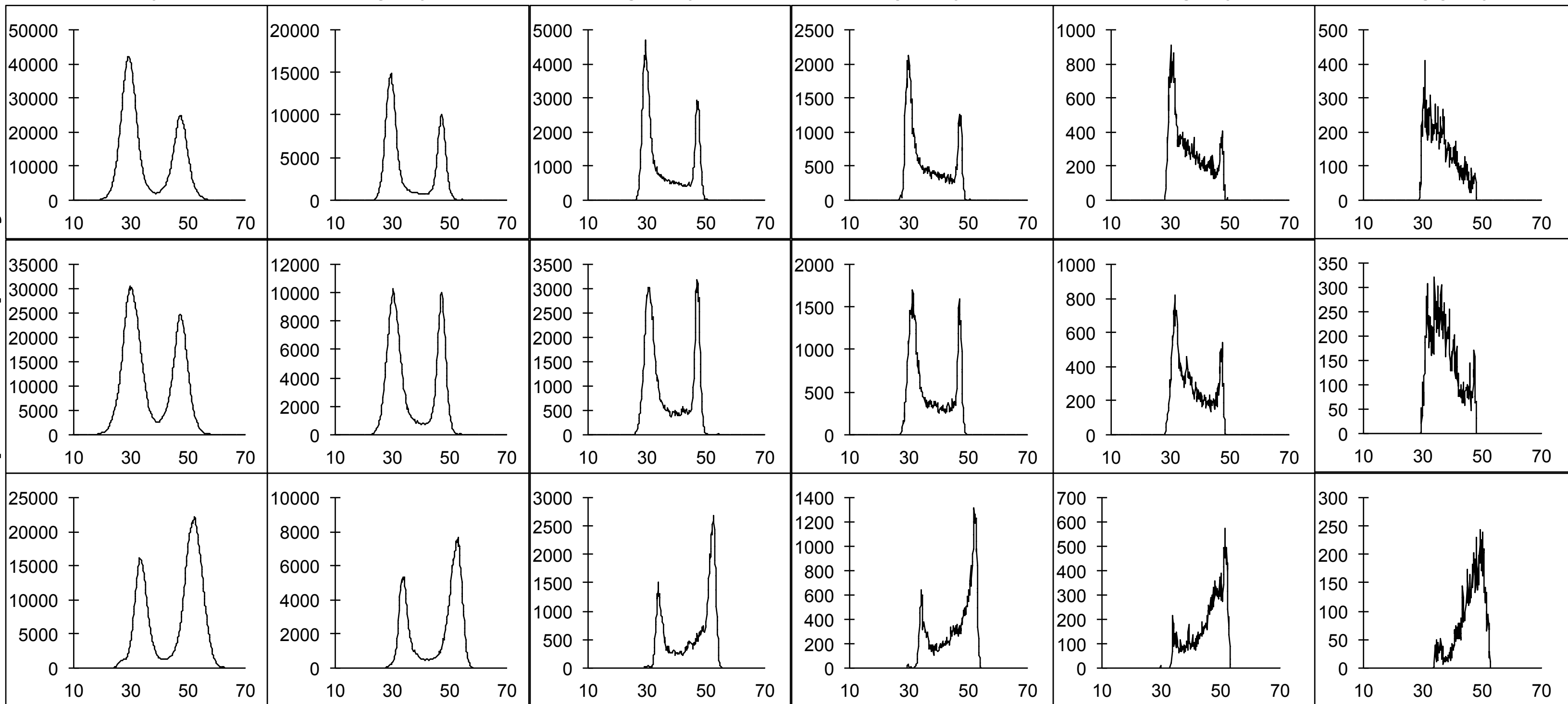

***Bd* ER-3**

***Lm* v23.1.3**

**Percent of guanine + cytosine (%GC)**

Supplement: S4 Fig — Window sizes included 2, 8, 32, 64, 128 and 256 kb. Step size was 1/128 of the window size. The bin size of the histograms is approximately 0.1% GC. Horizontal axes show GC percent and vertical axes show relative frequencies. (PDF) [file pgen.1005493.s004.pdf]

Sequence frequency

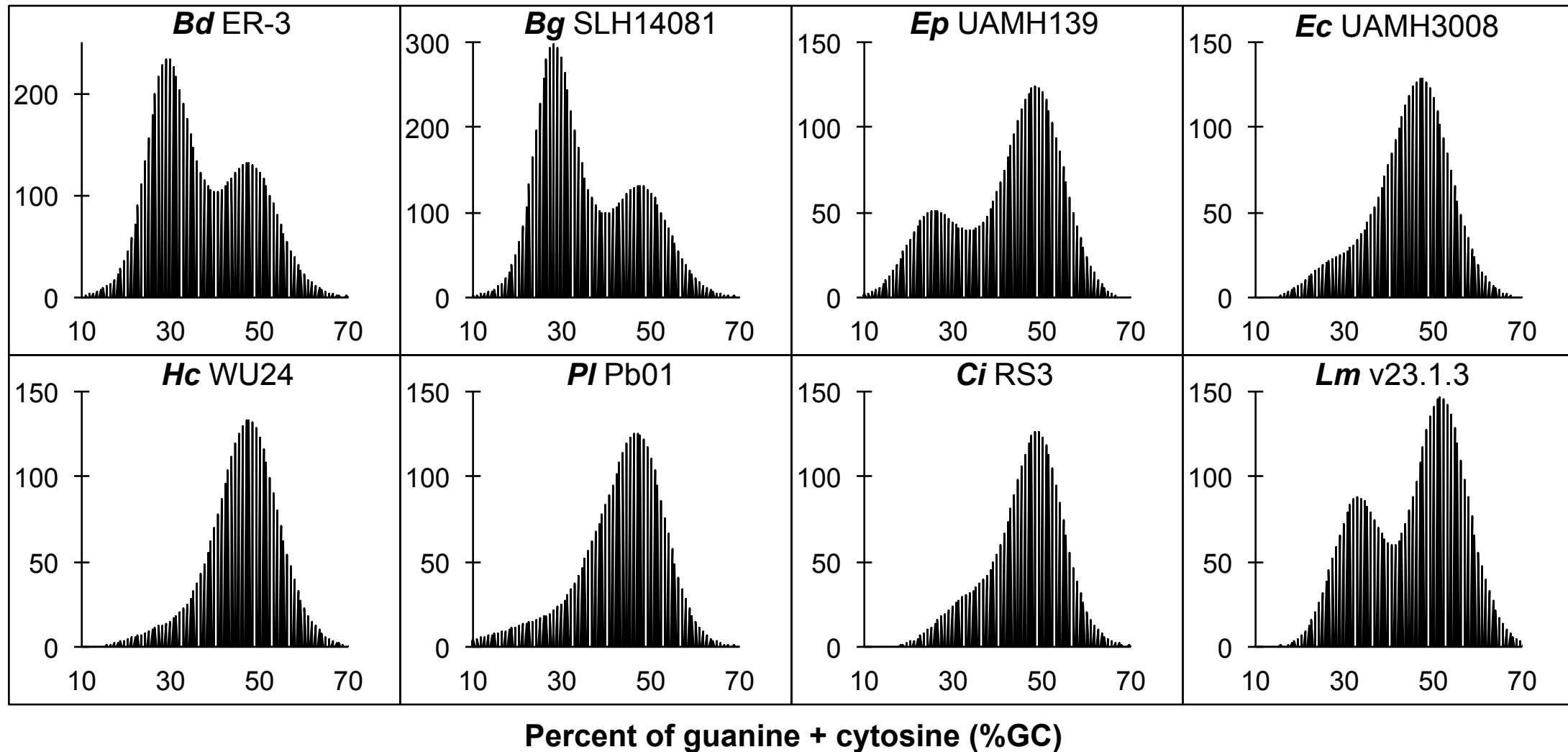

Supplement: S5 Fig — (PDF) [file pgen.1005493.s005.pdf]

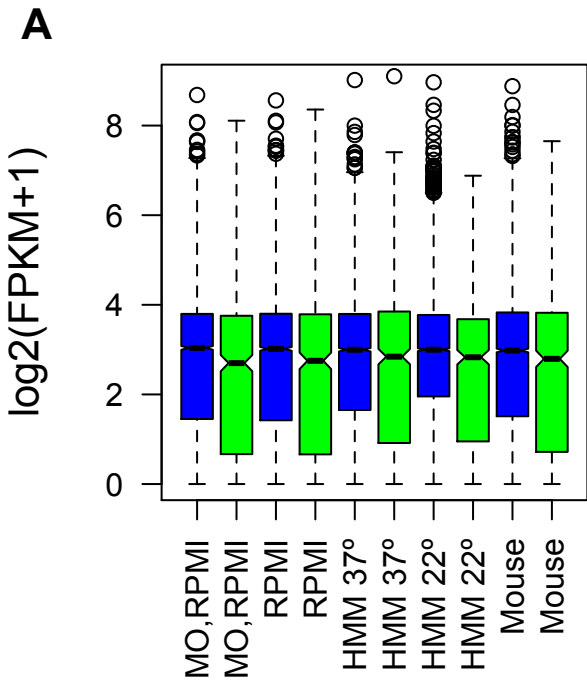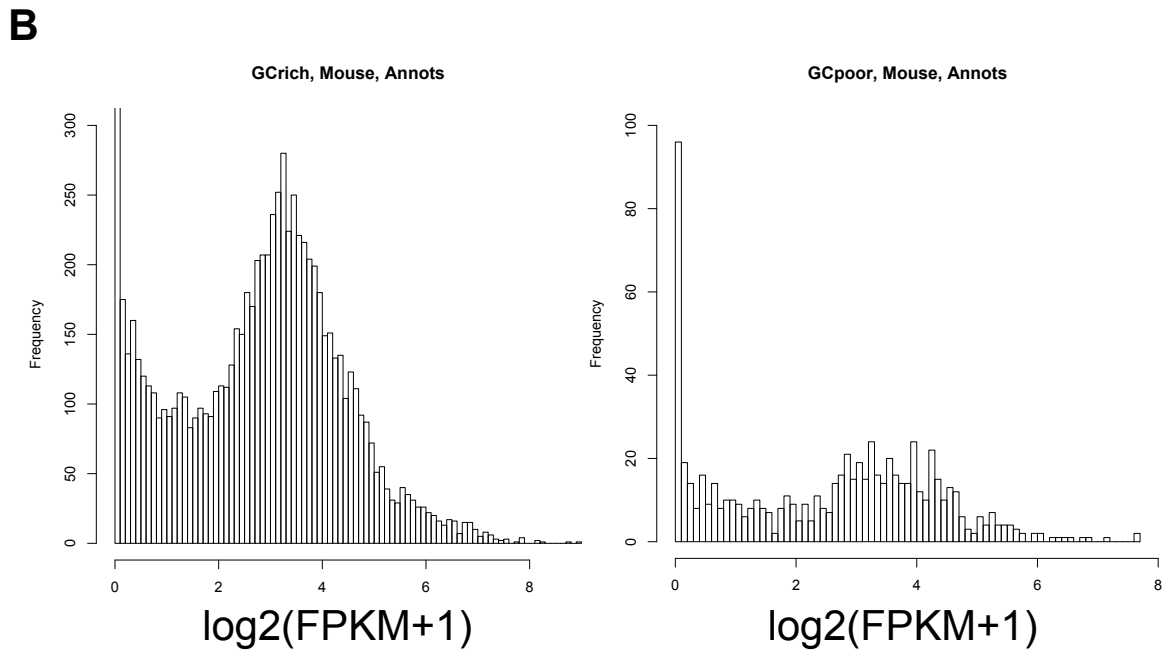

Supplement: S7 Fig — (A) Box plot of the gene expression (log2(FPKM+1)) of the genes located in GC-rich regions (blue) and genes located in GC-poor regions (green) in all five conditions of the RNA-Seq experiment of B. dermatitidis strain ATCC26199. Histograms in (B) show in the x-axis the distribution of the gene expression (log2(FPKM+1)) of those genes according their location during mouse infection. Similar distribution was observed in the other four conditions. (PDF) [file pgen.1005493.s007.pdf]

A

- *B. gilchristii* SLH14081
- *B. dermatitidis* ER-3
- *E. parva*
- Rebase (\*)

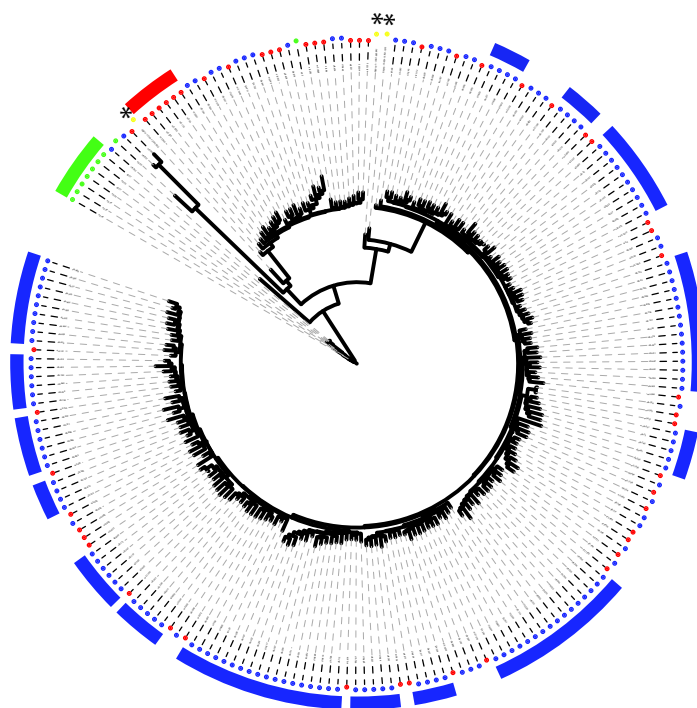

B

- *B. gilchristii* SLH14081
- *B. dermatitidis* ER-3

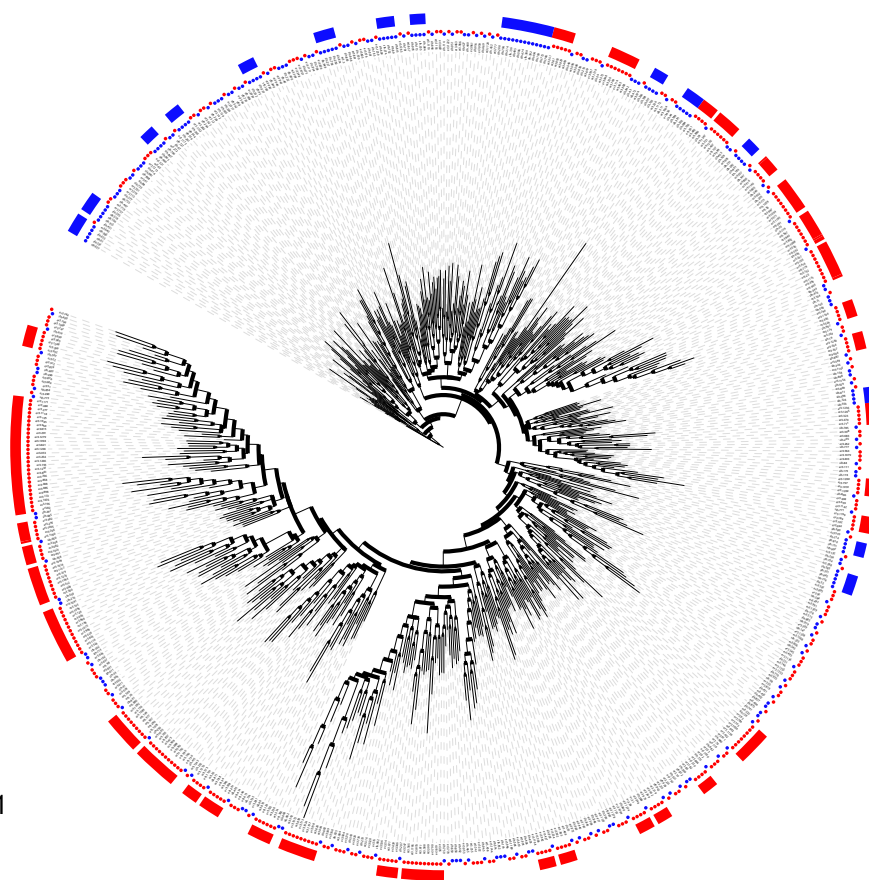

Supplement: S9 Fig — Four divergent clades of gypsy elements (A, B, Fig 2B and 2C) were identified from a phylogeny inferred using FastTreeDP from alignments of reverse transcriptase domains identified in gypsy elements of B. dermatitidis (ER-3), B. gilchristii (SLH14081) and E. parva (UAMH139). Each of the four clades is shown separately; A. Subgroup of 220 sequences includes non-ACa Repbase elements. B. Subgroup of 554 sequences specific to Blastomyces. The outer circle indicates strain specific duplications of four or more sequences. (PDF) [file pgen.1005493.s009.pdf]

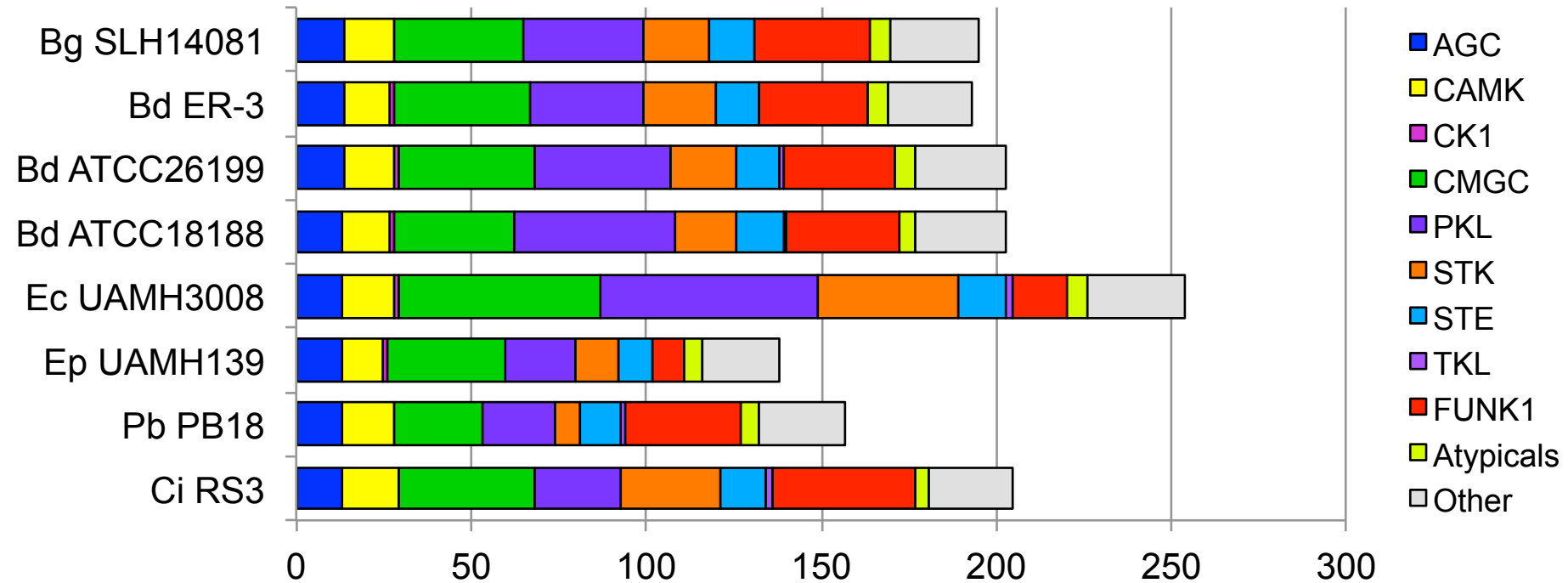

Supplement: S10 Fig — The kinomes of Blastomyces gilchristii (Bg; SLH14081) and B. dermatitidis (Bd; ER-3, ATCC26199 and ATCC18188) were compared with Emmonsia parva (Ep; UAMH139), E. crescens (Ec; UAMH3008), Paracoccidioides brasiliensis (Pb; Pb18) and Coccidioides immitis (Ci; RS3). Kinases are classified into major groups shown as colored blocks. Abbreviations: AGC, protein kinases A; CAMK, calcium/calmodulin-dependent kinases; CK1, casein kinase 1; CMGC, cyclin-dependent kinases (CDK), mitogen-activated, glycogen-synthase, and CDK-like kinases; STE, sterile phenotype kinases; FunK1, fungal-specific kinase 1; PKL, protein kinase subdomain-containing proteins; STK, serine/threonine protein kinase; STE, sterile phenotype kinases; TKL, tyrosine kinases. (PDF) [file pgen.1005493.s010.pdf]

Mouse

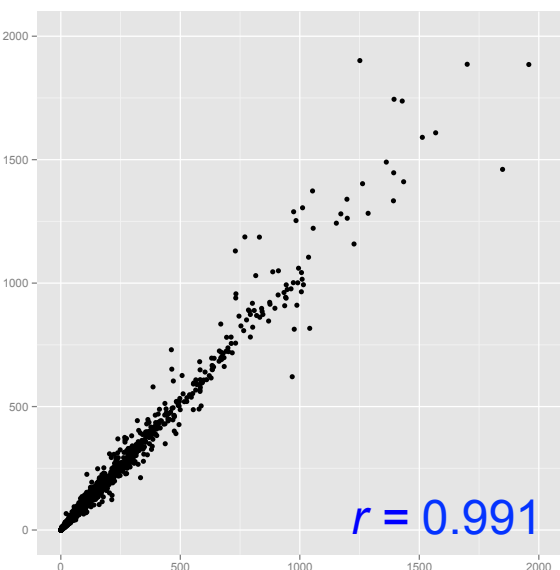

Yeast-M $\Phi$ -RPMI

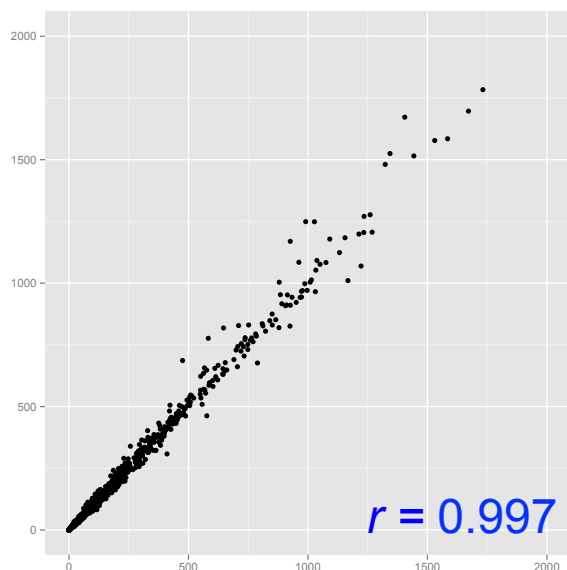

Yeast-RPMI

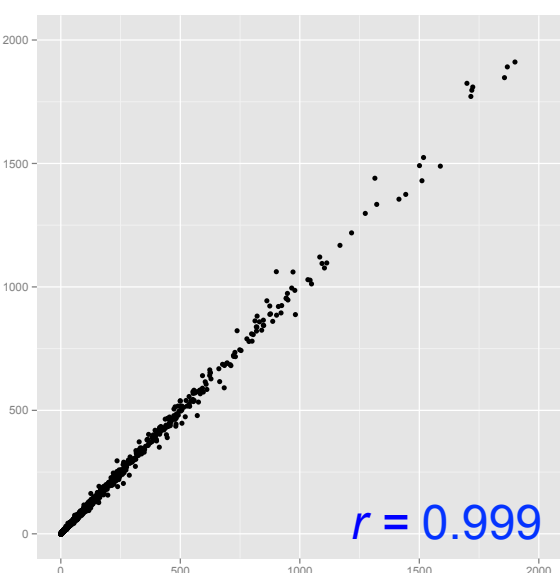

Yeast-HMM

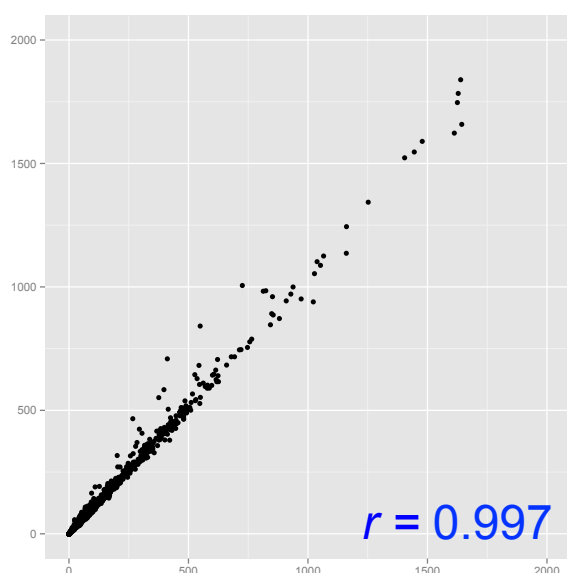

Mycelia-HMM

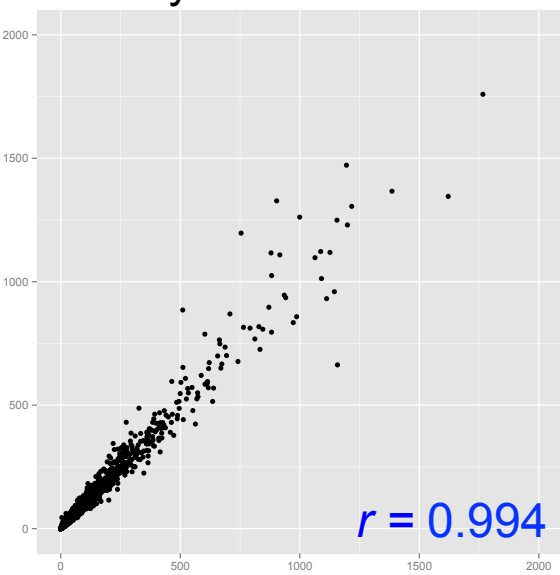

Supplement: S11 Fig — Two biological replicates for each condition of the RNA-Seq of Blastomyces dermatitidis (ATCC26199). (PDF) [file pgen.1005493.s011.pdf]
