# Supplementary material for: The Dynamic Genome and Transcriptome of the Human Fungal Pathogen Blastomyces and Close Relative Emmonsia
Source: PLoS Genet. 2015 Oct 6;11(10):e1005493. doi: 10.1371/journal.pgen.1005493 (PMC4595289; doi:10.1371/journal.pgen.1005493)
Supplement: S2 Fig — The percent coverage of genes with significant Blast similarity is shown for alignments above and below the recommended 70% coverage threshold; matches with less than 70% coverage suggest these are partial gene structures. (PDF) [file pgen.1005493.s002.pdf]

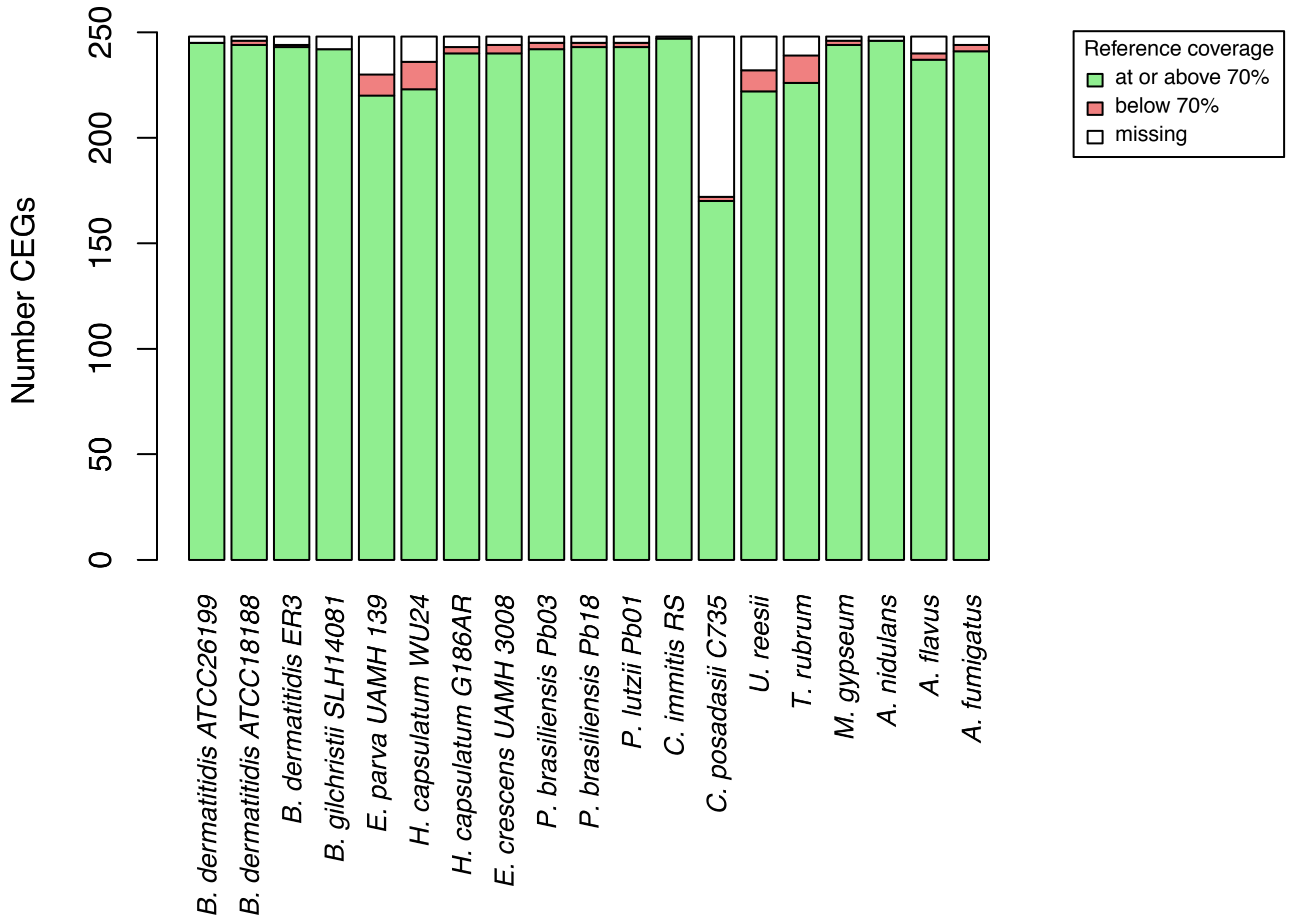
Number CEGs
0
50
100
150
200
250
Reference coverage
at or above 70%
below 70%
missing
B. dermatitidis ATCC26199
B. dermatitidis ATCC18188
B. dermatitidis ER3
B. gilchristii SLH14081
E. parva UAMH 139
H. capsulatum WU24
H. capsulatum G186AR
E. crescens UAMH 3008
P. brasiliensis Pb03
P. brasiliensis Pb18
P. lutzii Pb01
C. immitis RS
C. posadasii C735
U. reesii
T. rubrum
M. gypseum
A. nidulans
A. flavus
A. fumigatus
