# Supplementary material for: The Dynamic Genome and Transcriptome of the Human Fungal Pathogen Blastomyces and Close Relative Emmonsia
Source: PLoS Genet. 2015 Oct 6;11(10):e1005493. doi: 10.1371/journal.pgen.1005493 (PMC4595289; doi:10.1371/journal.pgen.1005493)
Supplement: S6 Fig — The example illustrates the intraspecific variability in presence/absence of GC-poor segments or ‘inserts’ and, even where their presence and location are conserved, the variability in their lengths. In (A) the dotplot of one complete scaffold of E. parva aligned to B. gilchristii strain SLH14081 (top) and B. dermatitidis strain ER-3 (bottom). In (B) the corresponding location of the inserts and the length; only insertion sites that were >15 kb for at least one strain are shown. This 265 kb region of the E. parva genome, lacks intermediate-sized (>15 kb) or long inserts, allowing its use as a simple reference for marking positions. (PDF) [file pgen.1005493.s006.pdf]

A

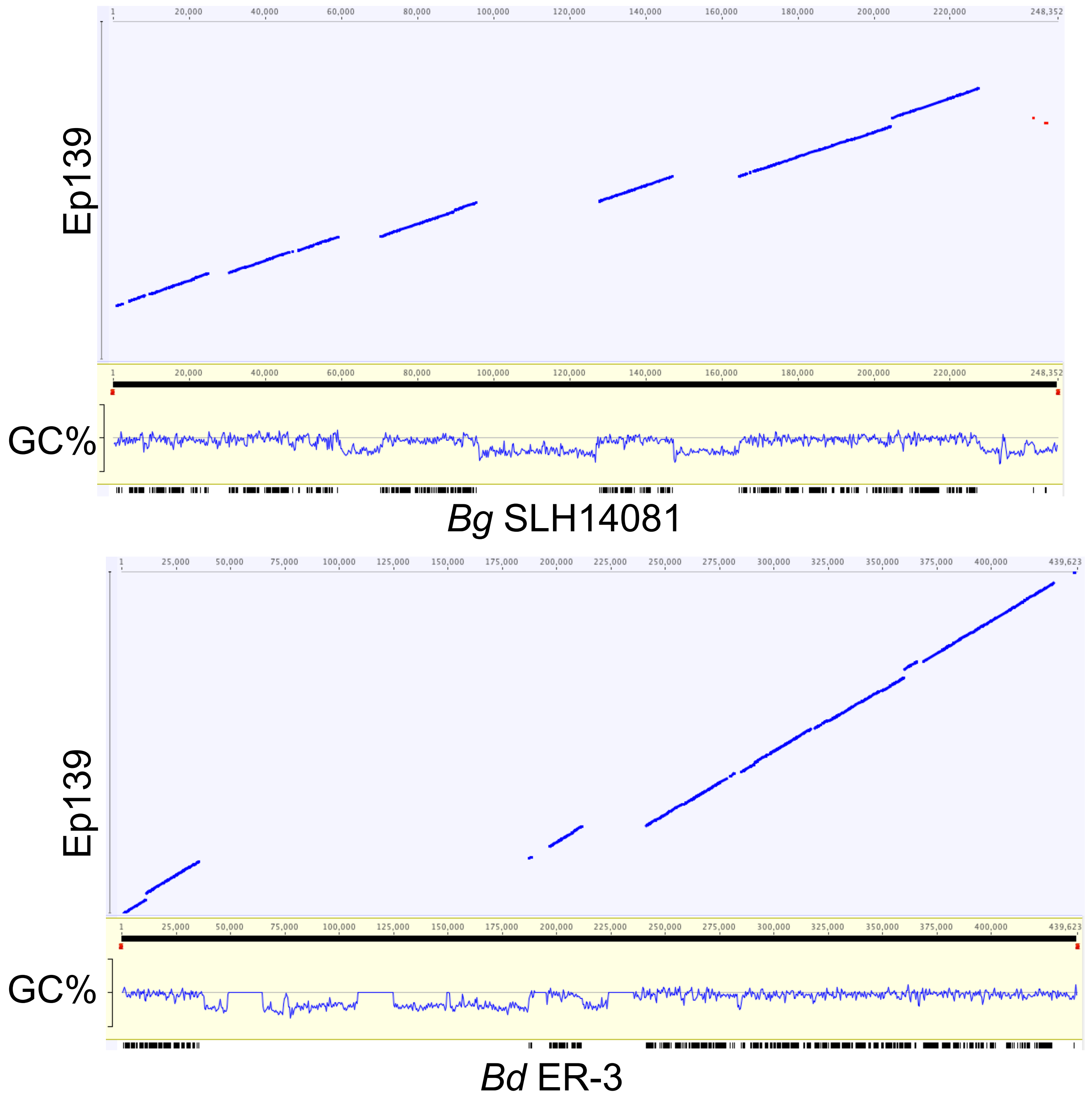

B

Strain	Supercontig	Length 1 ~42 kb	Length 2 ~68 kb	Length 3 ~96 kb	Length 4 ~124 kb	Length 5 ~144 kb
Bg SLH14081	19	>114	5	11	32	18
Bd ER-3	1	168	5	38	-	-
Bd ATCC26199	5	137	5	-	8	-
Bd ATCC18188	12	150	29	-	-	-
Ep UAMH139	242	-	-	-	-	-
