# Supplementary material for: The Dynamic Genome and Transcriptome of the Human Fungal Pathogen Blastomyces and Close Relative Emmonsia
Source: PLoS Genet. 2015 Oct 6;11(10):e1005493. doi: 10.1371/journal.pgen.1005493 (PMC4595289; doi:10.1371/journal.pgen.1005493)

Gypsy-1-I_ACa-int	2680
Gypsy-1-LTR_ACa	1179
Gypsy-1_ARO-I-int	841
Gypsy-47_MLP-I-int	607
Gypsy-67_MLP-I-int	361
Gypsy-7_SCH-I-int	311
Gypsy-87_MLP-I-int	225
Gypsy-1-I_AN-int	205
Gypsy-5_PPM-I-int	114
REALAA_I-int	95
Gypsy-93_MLP-I-int	94
Gypsy-3_GDe-I-int	91
Gypsy-76_MLP-I-int	86
Gypsy-6_MLP-I-int	85
Gypsy-19_MLP-I-int	76
Gypsy-35_MLP-I-int	73
Gypsy-9_LBS-I-int	66
TCN1-I-int	58
Gypsy-38_MLP-I-int	56
Gypsy-1_CCO-I-int	50

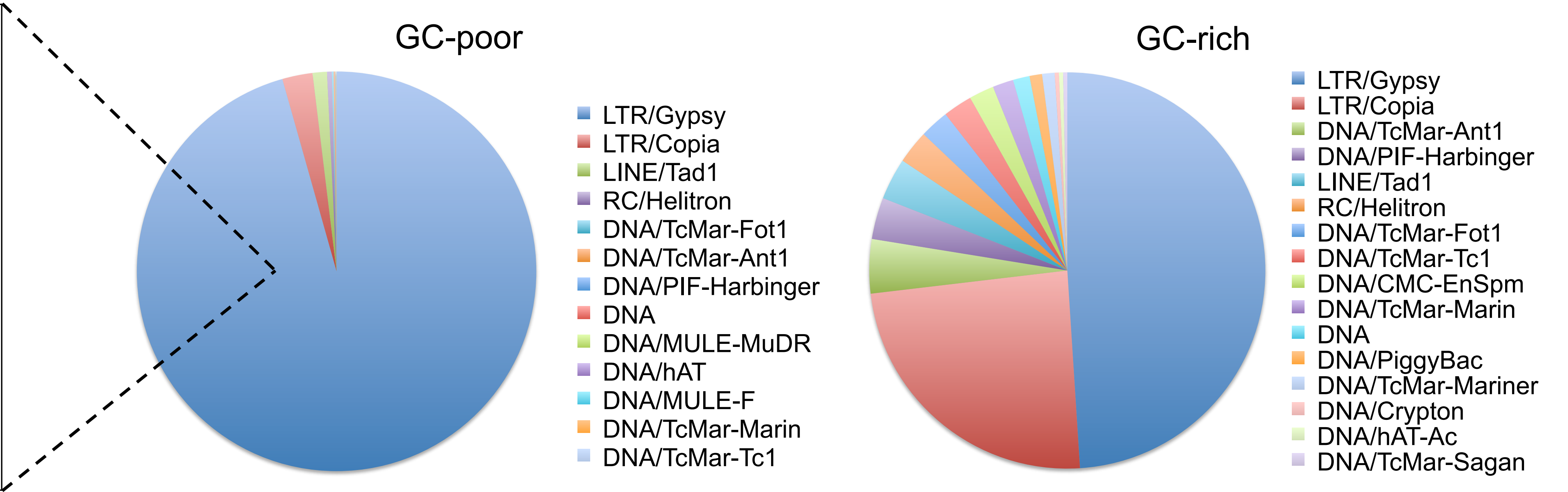

Supplement: S8 Fig — The list in the left box represent the first 20 LTR/Gypsy representing approximately 90% of the LTR/Gypsy family in the GC-poor regions. (PDF) [file pgen.1005493.s008.pdf]
