# Supplementary material for: The Dynamic Genome and Transcriptome of the Human Fungal Pathogen Blastomyces and Close Relative Emmonsia
Source: PLoS Genet. 2015 Oct 6;11(10):e1005493. doi: 10.1371/journal.pgen.1005493 (PMC4595289; doi:10.1371/journal.pgen.1005493)
Supplement: S1 Table — (DOCX) [file pgen.1005493.s013.docx]

**Table S1.** Phenotypic differences observed among *Blastomyces*, *E. parva* and *E. crescens*.

|  | ***E. parva*** | ***E. crescens*** | ***Blastomyces*** |
| --- | --- | --- | --- |
| Mammalian niche | e.g., rodents | e.g., rodents | incl. human |
| Agent of | adiaspiromycosis | adiaspiromycosis | blastomycosis |
| Inhaled form | conidia | conidia | conidia |
| Pathogenic form | adiaspores* | adiaspores | yeast |
| Transition temp. | 40 ºC | 35-37 ºC | 37 ºC |
| Adiaspore size *in vitro* | 25 (10-20) μm | 120 (200-480) μm | Not applicable |
| Thick adiospore wall | 2-4 μm | 10-80 μm | Not applicable |
| Adiapore size *in vivo* | up to 46 μm | up to 700** μm | Not applicable |
| Nuclei in adiaspores | 1 or several | numerous | Not applicable |
| Cell gigantism *in vivo* | yes | yes | rare |

Adapted from [1,2]. *: adiaspores, also called adiaconidia, are defined as a fungal spore enlarging without multiplication at elevated temperature. **: adiaspore size appears to depend on rodent host species, e.g.: size in *Pitmys subterraneus* < in *Apodemus sylvatica* < in *Clethriomys glareolus*

1. Hejtmánek M. Dimorphism in *Chrysosporium parvum*. In: Szaniszlo PJ, Harris JL, editors. Fungal Dimorphism. Springer US; 1985. pp. 237–261. Available: http://link.springer.com/chapter/10.1007/978-1-4684-4982-2_10

2. De Hoog GS, Guarro J, Gené J, Figueras MJ. Atlas of clinical fungi. 2000; viii + 1126 pp.
