# Supplementary material for: The Dynamic Genome and Transcriptome of the Human Fungal Pathogen Blastomyces and Close Relative Emmonsia
Source: PLoS Genet. 2015 Oct 6;11(10):e1005493. doi: 10.1371/journal.pgen.1005493 (PMC4595289; doi:10.1371/journal.pgen.1005493)
Supplement: S2 Table — (DOCX) [file pgen.1005493.s014.docx]

**Table S2.** Optical map information for *Blastomyces* strain SLH14081.

| **Optical Linkage Group** | **Estimated Size (Mb)** | **Mapped Scaffolds** | **Scaffold Size (Mb)** |
| --- | --- | --- | --- |
| contig21 | 3.690 | 4, 13 | 6.282 |
| contig23 | 5.548 | 10,25,37,17,28, 45 | 5.593 |
| contig24 | 3.728 | 20,23 | 2.044 |
| contig123 | 0.730 | 30 | 0.572 |
| contig467 | 1.617 | 21,33,34 | 1.779 |
| contig736 | 1.990 | 5 | 4.119 |
| contig981 | 3.807 | 7,24,41 | 4.176 |
| contig1169 | 4.830 | 11,29 | 2.614 |
| contig1275 | 4.786 | 16,6 | 5.116 |
| contig1280 | 4.009 | 1 | 8.167 |
| contig1305 | 3.941 | 2 | 7.091 |
| contig1399 | 4.498 | 3 | 6.423 |
| contig1400 | 5.765 | 14 | 1.675 |
| contig1506 | 5.867 | 48,31,22, 47 | 1.646 |
| contig1611 | 9.728 | 12,32,15,8,19,26 | 8.601 |
| **Total** | **64.534** |  | **65.890** |
