# Supplementary material for: The Dynamic Genome and Transcriptome of the Human Fungal Pathogen Blastomyces and Close Relative Emmonsia
Source: PLoS Genet. 2015 Oct 6;11(10):e1005493. doi: 10.1371/journal.pgen.1005493 (PMC4595289; doi:10.1371/journal.pgen.1005493)
Supplement: S7 Table — (DOCX) [file pgen.1005493.s019.docx]

**Table S7.** Secondary metabolite gene clusters predicted by Antismash.

| Genome* | NRPS | PKS | NRPS-PKS | Terpene | Terpene-nrps | Lantipeptide | Other | Total |
| --- | --- | --- | --- | --- | --- | --- | --- | --- |
| *Bd* ER-3 | 3 | 1 | 0 | 5 | 1 | 1 | 4 | 15 |
| *Bg* SLH14081 | 3 | 1 | 0 | 5 | 1 | 1 | 4 | 15 |
| *Ep* UAMH139 | 3 | 4 | 0 | 6 | 1 | 1 | 4 | 19 |
| *Hc* WU24 | 4 | 2 | 0 | 4 | 1 | 0 | 4 | 15 |
| *Ec* UAMH 3008 | 3 | 2 | 0 | 4 | 1 | 1 | 5 | 16 |
| *Pb* Pb18 | 4 | 3 | 0 | 4 | 0 | 1 | 5 | 17 |
| *C. immitis* | 5 | 8 | 2 | 5 | 0 | 1 | 3 | 24 |
| *U. reesii* | 6 | 5 | 3 | 5 | 0 | 0 | 2 | 21 |
| *T. rubrum* | 12 | 10 | 2 | 9 | 0 | 0 | 3 | 36 |
| *Abbreviations: *Bd*, *Blastomyces dermatitidis*; *Bg*, *B. gilchristii*; *Ep*: *Emmonsia parva*; Ec: *E. crescens*; *Hc*, *Histoplasma capsulaum*; *Pb*, *Paracoccidioides brasiliensis*. | | | | | | | | |
