# Supplementary material for: The Dynamic Genome and Transcriptome of the Human Fungal Pathogen Blastomyces and Close Relative Emmonsia
Source: PLoS Genet. 2015 Oct 6;11(10):e1005493. doi: 10.1371/journal.pgen.1005493 (PMC4595289; doi:10.1371/journal.pgen.1005493)
Supplement: S12 Table — (DOCX) [file pgen.1005493.s024.docx]

**Table S12**. RNA-Seq mapping statistics.

| **Condition** | **RSEM alignments** | **Total reads** | **% Aligned** |
| --- | --- | --- | --- |
| Mycelia-HMM1 | 55625194 | 61235346 | 90.84% |
| Mycelia-HMM2 | 58501966 | 64065980 | 91.32% |
| *in vivo*-Mouse1 | 53438996 | 66298414 | 80.60% |
| *in-vivo*-Mouse2 | 51626120 | 66088832 | 78.12% |
| Yeast-HMM1 | 60711408 | 68499460 | 88.63% |
| Yeast-HMM2 | 55874316 | 63053188 | 88.61% |
| Macrophages-RPMI1 | 54766974 | 63749608 | 85.91% |
| Macrophages-RPMI1 | 56067434 | 64331760 | 87.15% |
| Yeast-RPMI1 | 58332464 | 67328346 | 86.64% |
| Yeast-RPMI2 | 58723728 | 67098146 | 87.52% |
