# Supplementary material for: The Dynamic Genome and Transcriptome of the Human Fungal Pathogen Blastomyces and Close Relative Emmonsia
Source: PLoS Genet. 2015 Oct 6;11(10):e1005493. doi: 10.1371/journal.pgen.1005493 (PMC4595289; doi:10.1371/journal.pgen.1005493)
Supplement: S15 Table — (DOCX) [file pgen.1005493.s027.docx]

**Table S15.** Genomes of Onygenales and *Aspergillus* species compared in this study.

| **Species** | **Size (Mb)** | **Strains** |
| --- | --- | --- |
| *Blastomyces dermatitidis* | 65-69 | ER-3, ATCC26199, ATCC18188 |
| *Blastomyces gilchristii* | 75 | SLH14081 |
| *Emmonsia parva* * | 31^#^ | UAMH139 |
| *Histoplasma capsulatum* | 28 | WU24, G186AR |
| *Emmonsia crescens* * | 31^#^ | UAMH3008 |
| *Paracoccidioides brasiliensis* | 30 | Pb03, Pb18 |
| *Paracoccidioides lutzii* | 33 | Pb01 |
| *Coccidioides immitis* | 29 | RS |
| *Coccidioides posadasii* | 27 | C735 delta |
| *Uncinocarpus reesii* * | 22 | 1704 |
| *Microsporum gypseum* | 23 | CBS 118893 |
| *Trichophyton rubrum* | 23 | CBS 118892 |
| *Aspergillus nidulans *** | 30 | FGSC A4 |
| *Aspergillus flavus* | 37 | NRRL3357 |
| *Aspergillus fumigatus* | 29 | Af293 |

* typically not pathogenic in humans, except in cases of reduced immunocompetence.

** only noted in the environment.

^#^ genome size estimates are 45-55 for *E. parva* and 35-40 Mb for *E. crescens.*
