# Supplementary material for: The Dynamic Genome and Transcriptome of the Human Fungal Pathogen Blastomyces and Close Relative Emmonsia
Source: PLoS Genet. 2015 Oct 6;11(10):e1005493. doi: 10.1371/journal.pgen.1005493 (PMC4595289; doi:10.1371/journal.pgen.1005493)
Supplement: S1 Text — Possible biological meaning of the GC-poor tracts; Functional enrichment of genes in GC-poor tracts; The GATA transcription factor SREB and siderophore use; Secondary metabolite biosynthesis clusters; Characterization of gypsy element expansion; Gene expression changes in amino acid metabolism. (DOCX) [file pgen.1005493.s028.docx]

**S1 Text for Munoz et al. “The dynamic genome and transcriptome of the human fungal pathogen *Blastomyces* and close relative *Emmonsia*”**

**List of Supplementary Notes**

Possible biological meaning of the GC-poor tracts.

Functional enrichment of genes in GC-poor tracts.

The GATA transcription factor *SREB* and siderophore use.

Secondary metabolite biosynthesis clusters.

Characterization of gypsy element expansion.

Gene expression changes in amino acid metabolism.

**Supplementary Notes**

**Possible biological meaning of the GC-poor tracts**

At first sight, it might appear that the GC-poor stretches of *Blastomyces* are simply ‘junk’, ‘selfish’, or ‘parasitic’ DNA that has practically no benefits for the fungus, i.e., that this fungus has just been less successful or conscientious in keeping its junk DNA at low levels than the phylogenetically close genera *Histoplasma* and *Paracoccidioides*. The success of *Blastomyces* as a pathogen might then indicate that, although its genome size is now roughly twice that of some of its phylogenetic neighbors, the fungus has reached an equilibrium that is evolutionarily stable, albeit with a sizable amount of junk that the fungus has to maintain. Conceivably, loss of a mechanism of transposition or transposon control in a lineage leading to *Blastomyces* could have allowed the initial spreading of largely repetitive tracts across the genome.

Another possible hypothesis could have been that the two distinct GC ranges observed in the sequenced strains of *Blastomyces* (both containing fungal genes), separated by a zone around 38% GC that is almost devoid of DNA, could correspond to two different, broadly defined states of chromatin, with the GC-poor stretches favoring more closed chromatin or heterochromatin conformations that would be compatible with reduced or only context-dependent expression. The differential enrichments we observed, in our presence/absence analyses of *Blastomyces* versus other Ajellomycetaceae, of GO terms related to nucleosome assembly (GO:0000786, GO:0006334) could be compatible with chromatin conformations. In mammalian genomes, for example, GC-poor DNA is often (as a time average during interphase, and/or as a space average across tissues and cell types of the body) wrapped into heterochromatin or closed chromatin. Such chromatin states contrast with the more open chromatin configurations of mammalian GC-rich DNA [1] surrounding, for example, housekeeping genes or other CpG island genes [2,3]. Indeed, most genes in the GC-poor, gene-poor regions of the human genome [4,5] are tissue- or cell type-specific, (e.g., developmental genes), and the preferentially closed chromatin in such regions tends to make transcriptional activation more difficult by essentially requiring additional obstacles to be overcome [6].

Chromatin states and their changes have been investigated in fungi in model organisms such as *Candida albicans* (e.g., in the context of the white-opaque phase transition, [7]), but few if any experimental studies have explicitly addressed the variable chromatin component of gene regulation in Onygenales fungi*.* It would have been a reasonable speculation that the GC-poor DNA in *Blastomyces* could have a considerably more closed chromatin structure than the GC-rich DNA of the same genome. The chromatin difference, i.e., compartmentalized organization of genes, could then have given those genes that are programmed to be used only under specific, rare conditions an additional security against ectopic transcriptional activation, as their unprogrammed transcription might often be detrimental to the fungus or at least squander resources of the cell. In mammals, such preference for closed chromatin is seen also in parasitic integrated DNA with a latent period in its life cycle; for example, in the case of HIV and other mammalian lentiviruses, stably integrated proviruses tend to be preferentially located in closed-chromatin, GC-poor regions of their host genome (unlike oncoviruses) [8–10]. Locking down certain genes into closed chromatin or heterochromatin compartments might therefore conceivably be an effective way of controlling or limiting their transcription.

In *B. dermatitidis*, the expression data we report do not appear to support such a general notion of genome-wide compartmentalization of transcription via mosaics of long tracts having different chromatin states. Indeed, we did not find consistently lower, or more obviously phase-specific, expression in the GC-poorer regions/tracts of this genome. Nevertheless, we noted some well-studied fungal genes that are located within or near the borders of GC-poor regions, where in principle transcription could in some phase(s) be affected by embedding or adjacent DNA that is transiently in heterochromatin or closed chromatin states. Such genes include, for example, the *Blastomyces* yeast-phase specific gene (*BYS1*)*,* which has been studied as a monitor of transitions from and to yeast [11,12]. In principle, a regulatory or biochemical chain of events might be reliably blocked by keeping in the closed- or heterochromatin of GC-poor DNA just one or two critical genes of that chain. However, a possible danger of ‘keeping’ a virulence or other gene in such an unstable GC-poor, repeat-rich region is that it might get lost, and that variants without the gene might get fixed in a population and require a possibly rare transfer event in order to regain the gene.

The involvement of chromatin states and heterochromatin as a part of virulence strategies is not a new idea (see [13], or the HIV latency example mentioned above). Although our expression results do not give support to such genome-wide involvement in *B. dermatitidis*, different chromatin states may be involved in regulating programmed expression of certain genes in certain phase(s).

A more detailed gene-by-gene analysis of the roles of genes found in GC-poor tracts might reveal whether or not some or most of those tracts could coincide with the notion of ‘accessory’ or ‘dispensable’ chromosome segments [14], where the presence/absence variation and/or location instability might have a net advantage, despite a possible risk of gene loss.

Whether GC bimodality is a feature of all *Blastomyces* genomes is unknown. A previous study used thermal denaturation curves to identify strain-to-strain variation of the presence of GC bimodality [15]. However, as the authors also detected GC bimodality in *Histoplasma*, which is clearly shown here not to be the case, their findings may highlight a different phenomenon than large-scale GC heterogeneity within nuclear chromosomes. Further sequencing of *Blastomyces* strains could shed light on this question.

**Functional enrichment of genes in GC-poor tracts**

Overall, genes in GC-poor regions showed modest enrichment for functional categories, including a M1 aminopeptidase family (PF01433, PF13485) in both ER-3 and SLH14018. In addition some differences were identified between the two isolates; SLH14081 GC-poor regions have fewer than expected protein kinases (PF00069) and more chromatin-related genes based on GO terms, differences not found in ER-3 (**S5 Table**). In both genomes, predicted secreted proteins are not enriched in GC-poor regions, further supporting the general similarity of gene content in both GC-poor and other regions of the *Blastomyces* genome.

**The GATA transcription factor *SREB* and siderophore use**

Previous results of Gauthier et al. [16] provide evidence indicating that the siderophore synthesis repressor of *Blastomyces* (*SREB*, BDFG_05131) may be a global regulator, in the sense of regulating not only genes associated with siderophore use but also a number of different and apparently unrelated genes having a GATA binding site(s) in their regulatory region(s). If *SREB* is impaired so that it cannot exert its repressor function, *Blastomyces* is unable to revert from the yeast phase to the mycelial phase under standard transition/germination conditions, and under iron-replete conditions continues to produce siderophores.

As might be anticipated from the observation that GATA binding sites are GC-poor, we found that genes previously noted to have two or more potential GATA binding sites [16] are preferentially located in the long GC-poor tracts we delimited in this study. Shorter GC-poor stretches, identified by running IsoFinder with parameters 0.95 and 3 kb instead of 0.99 and 5 kb, also contained two GATA motif-containing genes that had shown dramatic expression differences in presence *vs.* absence of normal *SREB* gene function: acyl-CoA dehydrogenase (BDFG_05777; 4 GATA motifs), and glycosyl hydrolase/endo-1,3(4)-beta-glucanase (BDFG_03060; 1 GATA motif). In our transcriptomic analysis the *SREB* gene is significantly upregulated in yeast in HMM compared to infections of both macrophages and mouse. Since the *in vivo* conditions are iron-poor environments compared to *in vitro* culture, the transcription of *SREB* was decreased as expected. Of 422 genes that contain the upstream GATA sites(s) 28.4% were significantly upregulated in mouse infection, macrophage infection or yeast cells in HMM. Some of those genes include genes involved in the biosynthesis of siderophores such as *SID1* (BDFG_01074), in the transport of ornithine from the mitochondria into the cytosol such as *AMCA* (BDFG_01148), in the uptake of siderophores such as *MIRB* and *MIRC*, and they also include a bZIP transcription factor (*HAPX*, BDFG_04516), all of which were significantly upregulated in mouse infection. *HAPX* is indispensable for the transcriptional remodeling required for adaption to iron starvation in the opportunistic fungal pathogen *Aspergillus fumigatus* [17]. The gene peroxisomal dehydratase (BDFG_01073) that is part of the siderophore biosynthetic cluster is also highly induced during mouse infection; this gene also appears to be under the control of the *SREB* gene and contains upstream GATA binding sites.

**Characterization of gypsy element expansion**

To examine the dynamics of gypsy element expansion in *Blastomyces*, the phylogenetic relationship of elements from different species was examined. As individual copies identified by RepeatMasker had a large variation in size, precluding obtaining a high quality alignment, we selected out only the reverse transcriptase domains present in all gypsy copies. The PFAM RVT_1 domain was identified as described in the Methods; sequences were translated allowing for stop codons within the domain, as few sequences contained an intact open reading frame across this domain.

Phylogenetic analysis using FastTreeDP supported four major subdivisions of the gypsy elements identified. The largest included the RepBase ACa element and 943 additional sequences from *Blastomytces* (SLH14018 and ER-3) *and E. parva* (**Fig 4B**); on average these sequences share 77.8% identity at the protein level. Another subgroup was similarly diverse although smaller, and included three other RepBase elements from related fungi (Gypsy-1-I_AN_1, Gypsy-1_ARO-I_3, Gypsy-1_Cop-I_2) and 217 additional sequences from *Blastomyces* (SLH14018 and ER-3) *and E. parva* (**S9A Fig**). On average these sequences shared 64.7% identity at the protein level. Two subgroups specific to *Blastomyces* were identified, one of 544 sequences (**Fig 4C**) and one of 623 sequences (**S9B Fig**); elements in these groups shared 83.3% and 76.9% identity respectively.

**Secondary metabolite biosynthesis clusters**

To arrive at a first notion of the extent to which the GC-poor tracts, or shorter GC-poor regions, in *Blastomyces* or *Emmonsia* might harbor previously characterized clusters of virulence-related or other ‘production genes’, we identified predicted biosynthetic clusters using the program antiSMASH 2 (antibiotics and Secondary Metabolism Analysis SHell; [18]). In the *B. dermatitidis* assembly with the highest continuity, ER-3, 15 clusters were predicted, including one 251-kb cluster that included a type T1pks polyketide synthase (PKS) gene, three type Nrps (non-ribosomal peptide synthase) clusters, five terpene clusters, and one terpene-nprs cluster (**S7 Table**). The Nrps clusters were of particular interest; two correspond, respectively, to a cluster beginning with the gene for *sod3* that we had found to be upexpressed in *B. dermatitidis* during the interaction with macrophages and in the mouse model, and to the conserved siderophore biosynthesis gene cluster involving but not physically including *mirB*, which we had found to be upexpressed in *B. dermatitidis* in the mouse model, and further to be present in *E. parva* and *Blastomyces* but missing in *E. crescens*. The correspondence with long GC-poor tracts (< 38% GC) as defined in this study was only very partial: the long T1pks cluster included only one short 39-kb GC-poor tract that did not contain the PKS gene; and other clusters contained at most smaller subregions that belonged to the GC-poor tracts.

Although biosynthesis clusters do not systematically exhibit consistently low GC over long stretches, and in this sense are not likely to help explain the feature of long GC-poor tracts in *Blastomyces*/*Emmonsia*, they are of intrinsic interest from the viewpoints of natural product mining, evolutionarily conserved clustering or expression patterns, and changes of chromatin states. Indeed, experimental studies have addressed expression and roles of genes from the widely conserved siderophore cluster in the fungus *Aspergillus nidulans* and in *H. capsulatum* [19–22]. Comparing the gene conservation of *Blastomyces*, or its less pathogenic sister species *E. parva*, may offer new clues as to how efficient exogenous gene control might be achieved.

**Gene expression changes in amino acid metabolism**

Changes in amino acid metabolism were prevalent in both the macrophage co-cultured and *in vivo Blastomyces*, suggesting the recycling of amino acids as an energy source. Both in macrophages and *in vivo*, *Blastomyces* increased transcription of dehydrogenases involved with branched chain amino acid catabolism; this may facilitate the breakdown of leucine, isoleucine, and valine to acetyl-CoA that can be utilized as a carbon source. In *H. capsutalum*, leucine catabolism contributes to yeast survival in macrophages [23]. Surprisingly, transcript abundance for isocitrate lyase (*ICL* - BDFG_08653) exhibited a < 2-fold change. Prior research has demonstrated upregulation of *ICL*, which encodes an enzyme in the glycoxylate cycle, occurs when *P. brasiliensis, Penicillium marneffei*, and *C. albicans* are phagocytozed by macrophages (Derengowski et al., 2008; Lorenz and Fink 2001; Sun *et al.* 2014)*.* In fungi, 2-carbon sources such as acetyl-CoA can enter the TCA cycle via the glyoxylate pathway. When co-cultured with macrophages, isocitrate lyase was increased 1.4-fold (versus no macrophages), whereas transcription was increased 2.4 – 3.2-fold during pulmonary infection (versus macrophages and no macrophages). Increased transcript abundance *in vivo* infection suggests the potential for the glyoxylate pathway to contribute to pathogenesis. In *Penicillium marneffei* and *C. albicans*, isocitrate lyase transcription increases during internalization by macrophages and contributes to virulence [24,25].

Cysteine degradation by cysteine dioxygenase (BDFG_08059) to L-cysteinesulfinic acid, which can be used for taurine biosynthesis or metabolized to pyruvate and sulfite, may contribute to *B. dermatitidis* pathogenesis. In *C. albicans,* cysteine dioxygenase-1 (*CDG1*) is co-regulated with a sulfite efflux pump (*SSU1*). Similarly, homologs of *CDG1* and *SSU1* are highly upregulated in *B. dermatitidis* during *in vivo* infection. In mice, deletion of *CDG1* in *C. albicans* results in attenuated virulence [26]. Exported sulfite can destabilize host proteins by reducing disulfide bonds and facilitate growth of dermatophytes on keratinized tissue [27]. Furthermore, the very specific increase in cysteine catabolism and biosynthesis during *in vivo* infection (versus macrophage co-culture) suggests that cysteine may be critical to virulence.

**Supplementary References**

1. Tazi J, Bird A. Alternative chromatin structure at CpG islands. Cell. 1990;60: 909–920.

2. Larsen F, Gundersen G, Lopez R, Prydz H. CpG islands as gene markers in the human genome. Genomics. 1992;13: 1095–1107.

3. Ponger L, Duret L, Mouchiroud D. Determinants of CpG islands: expression in early embryo and isochore structure. Genome Res. 2001;11: 1854–1860. doi:10.1101/gr.174501

4. Zoubak S, Clay O, Bernardi G. The gene distribution of the human genome. Gene. 1996;174: 95–102.

5. Lander ES, Linton LM, Birren B, Nusbaum C, Zody MC, Baldwin J, et al. Initial sequencing and analysis of the human genome. Nature. 2001;409: 860–921.

6. Beisel C, Paro R. Silencing chromatin: comparing modes and mechanisms. Nat Rev Genet. 2011;12: 123–135. doi:10.1038/nrg2932

7. Klar AJ, Srikantha T, Soll DR. A histone deacetylation inhibitor and mutant promote colony-type switching of the human pathogen Candida albicans. Genetics. 2001;158: 919–924.

8. Jordan A, Bisgrove D, Verdin E. HIV reproducibly establishes a latent infection after acute infection of T cells in vitro. EMBO J. 2003;22: 1868–1877. doi:10.1093/emboj/cdg188

9. Tsyba L, Rynditch AV, Boeri E, Jabbari K, Bernardi G. Distribution of HIV-1 in the genomes of AIDS patients. Cell Mol Life Sci CMLS. 2004;61: 721–726. doi:10.1007/s00018-003-3436-6

10. Zoubak S, Rynditch A, Bernardi G. Compositional bimodality and evolution of retroviral genomes. Gene. 1992;119: 207–213.

11. Burg EF, Smith LH. Cloning and characterization of bys1, a temperature-dependent cDNA specific to the yeast phase of the pathogenic dimorphic fungus *Blastomyces dermatitidis*. Infect Immun. 1994;62: 2521–2528.

12. Krajaejun T, Wüthrich M, Gauthier GM, Warner TF, Sullivan TD, Klein BS. Discordant influence of *Blastomyces dermatitidis* yeast-phase-specific gene BYS1 on morphogenesis and virulence. Infect Immun. 2010;78: 2522–2528. doi:10.1128/IAI.01328-09

13. Merrick CJ, Duraisingh MT. Heterochromatin-mediated control of virulence gene expression. Mol Microbiol. 2006;62: 612–620. doi:10.1111/j.1365-2958.2006.05397.x

14. Covert SF. Supernumerary chromosomes in filamentous fungi. Curr Genet. 1998;33: 311–319. doi:10.1007/s002940050342

15. Guého E, Leclerc MC, de Hoog GS, Dupont B. Molecular taxonomy and epidemiology of *Blastomyces* and *Histoplasma* species. Mycoses. 1997;40: 69–81.

16. Gauthier GM, Sullivan TD, Gallardo SS, Brandhorst TT, Vanden Wymelenberg AJ, Cuomo CA, et al. SREB, a GATA transcription factor that directs disparate fates in *Blastomyces dermatitidis* including morphogenesis and siderophore biosynthesis. PLoS Pathog. 2010;6: e1000846. doi:10.1371/journal.ppat.1000846

17. Schrettl M, Beckmann N, Varga J, Heinekamp T, Jacobsen ID, Jöchl C, et al. HapX-mediated adaption to iron starvation is crucial for virulence of *Aspergillus fumigatus*. PLoS Pathog. 2010;6: e1001124. doi:10.1371/journal.ppat.1001124

18. Blin K, Medema MH, Kazempour D, Fischbach MA, Breitling R, Takano E, et al. antiSMASH 2.0--a versatile platform for genome mining of secondary metabolite producers. Nucleic Acids Res. 2013;41: W204–212. doi:10.1093/nar/gkt449

19. Hwang LH, Mayfield JA, Rine J, Sil A. *Histoplasma* requires SID1, a member of an iron-regulated siderophore gene cluster, for host colonization. PLoS Pathog. 2008;4: e1000044. doi:10.1371/journal.ppat.1000044

20. Hwang LH, Seth E, Gilmore SA, Sil A. SRE1 regulates iron-dependent and -independent pathways in the fungal pathogen *Histoplasma capsulatum*. Eukaryot Cell. 2012;11: 16–25. doi:10.1128/EC.05274-11

21. Edwards JA, Chen C, Kemski MM, Hu J, Mitchell TK, Rappleye CA. *Histoplasma* yeast and mycelial transcriptomes reveal pathogenic-phase and lineage-specific gene expression profiles. BMC Genomics. 2013;14: 695. doi:10.1186/1471-2164-14-695

22. Gründlinger M, Gsaller F, Schrettl M, Lindner H, Haas H. *Aspergillus fumigatus* SidJ mediates intracellular siderophore hydrolysis. Appl Environ Microbiol. 2013;79: 7534–7536. doi:10.1128/AEM.01285-13

23. Isaac DT, Coady A, Van Prooyen N, Sil A. The 3-hydroxy-methylglutaryl coenzyme A lyase HCL1 is required for macrophage colonization by human fungal pathogen *Histoplasma capsulatum*. Infect Immun. 2013;81: 411–420. doi:10.1128/IAI.00833-12

24. Lorenz MC, Fink GR. The glyoxylate cycle is required for fungal virulence. Nature. 2001;412: 83–86.

25. Sun J, Li X, Feng P, Zhang J, Xie Z, Song E, et al. RNAi-mediated silencing of fungal acuD gene attenuates the virulence of *Penicillium marneffei*. Med Mycol. 2014;52: 167–178. doi:10.1093/mmy/myt006

26. Hennicke F, Grumbt M, Lermann U, Ueberschaar N, Palige K, Böttcher B, et al. Factors supporting cysteine tolerance and sulfite production in *Candida albicans*. Eukaryot Cell. 2013;12: 604–613. doi:10.1128/EC.00336-12

27. Grumbt M, Monod M, Yamada T, Hertweck C, Kunert J, Staib P. Keratin degradation by dermatophytes relies on cysteine dioxygenase and a sulfite efflux pump. J Invest Dermatol. 2013;133: 1550–1555. doi:10.1038/jid.2013.41
